# The Current Landscape of Remote Digital Symptom Monitoring for Patients With Lung Cancer: Scoping Review

**DOI:** 10.2196/83666

**Published:** 2026-03-24

**Authors:** Hao Huang, Naomi Takemura, Hammoda Abu-Odah, Shuhan Li, Ting Mao, Zengjie Ye, Janelle Yorke

**Affiliations:** 1 School of Nursing, The Hong Kong Polytechnic University, 11 Yuk Choi Road, Hung Hom, Kowloon, Hong Kong, China (Hong Kong), 852 27666396; 2 JC STEM Lab of Digital Oncology Care Enhancement (DOCE), The Hong Kong Jockey Club Charities Trust, Hong Kong, China (Hong Kong); 3 School of Nursing, Guangzhou Medical University, Guangzhou, China; 4 Division of Nursing, Midwifery and Social Work, Faculty of Biology, Medicine and Health, University of Manchester, Manchester, United Kingdom

**Keywords:** digital health, electronic patient-reported outcome measure, ePROM, lung cancer, symptom experience, scoping review, symptom monitoring

## Abstract

**Background:**

Remote digital symptom monitoring systems (rSMS) have been increasingly used in recent years to monitor symptoms, health-related quality of life, and other patient-reported outcomes in lung cancer. Previous studies have demonstrated variability in study design, types of rSMS, and outcomes used to assess benefits for patients and health care systems. However, there remains a lack of synthesized evidence pertaining to the similarities and differences among rSMS, including their theoretical underpinnings, key functional components, and reported benefits and limitations.

**Objective:**

This review aims to identify and synthesize existing research to map the current landscape of rSMS in lung cancer, including the theoretical foundations for its development and implementation, as well as its types, applications, and outcomes.

**Methods:**

This scoping review followed the Joanna Briggs Institute scoping review framework and adhered to the PRISMA-ScR (Preferred Reporting Items for Systematic Reviews and Meta-Analyses Extension for Scoping Reviews) guidelines. A comprehensive literature search was conducted from database inception to October 16, 2025, across 7 English-language databases and 3 Chinese-language databases (CNKI, WanFang, and SinoMed). Eligible studies were peer-reviewed original research articles examining rSMS among adults with lung cancer. Data were independently screened and extracted by 2 reviewers, with discrepancies resolved by a third reviewer. Quantitative data were extracted using a standardized form and synthesized descriptively. Content analysis was performed to analyze the qualitative data.

**Results:**

A total of 41 studies involving 11,765 patients and 85 health care providers were included. Twelve studies focused exclusively on advanced-stage lung cancer. Participants were generally middle-aged to older adults (mean ages 51‐74 y), with male participants typically comprising 30% to 50% across studies. Most studies were conducted in the United States (n=19). We identified 32 patient-reported outcome measures that were used either as core rSMS components or as study outcomes. Four common functional modules were observed across rSMS: data collection, data analysis, response systems, and patient education. Qualitative evidence was limited; the most frequently reported benefit was the promotion of patient-centered care. Health care providers raised concerns about uncertain effectiveness and increased workload.

**Conclusions:**

This scoping review highlights the promising role of rSMS in lung cancer care and provides a structured map of current evidence. It adds to prior literature in 3 ways. First, it summarizes how and how often theoretical frameworks are reported and applied in rSMS development and implementation. Second, it synthesizes and categorizes four common functional modules across systems. Third, it differentiates measures embedded as rSMS components from those used as evaluation outcomes. These contributions clarify current practices and methodological gaps and underscore the importance of theory-informed design, functional clarity, and stakeholder engagement in the development of patient-centered, clinically meaningful, and sustainable rSMS platforms.

## Introduction

Lung cancer remains the leading cause of morbidity and mortality in men and women worldwide [1,2]. Patients with lung cancer experience a range of debilitating symptoms, including dyspnea, hoarseness, persistent cough, and fatigue, all of which substantially diminish their health-related quality of life (HRQL) throughout the disease trajectory [3,4]. Consequently, identifying and monitoring these symptoms is critical to establishing a foundation for effective interventions and optimizing patient outcomes.

Patient-reported outcome measures (PROMs) have been adopted extensively to monitor and evaluate symptoms [3,5]. These are standardized questionnaires completed by patients to capture self-assessments of symptoms, HRQL, mental well-being, and functional status [6]. In recent years, PROMs have been largely recognized as highly valid and reliable instruments for assessing health care outcomes from the patient’s perspective [7-9]. Moreover, several guidelines have been established to facilitate the standardized use and enhance the effectiveness of electronic PROMs (ePROMs) in clinical practice [10-13].

Advancements in digital health technologies have facilitated the evolution of ePROMs, defined as tools for the remote, digital collection of patient-reported outcomes outside clinical settings [14,15]. The application of ePROMs overcomes traditional scalability limitations, including reliance on in-person data collection, manual workflows that delay reporting, and fragmented communication between patients and clinicians.

A variety of terms have been used to describe electronic symptom monitoring interventions, including, but not limited to, electronic symptom self-reporting systems [16], remote symptom monitoring systems [17], electronic patient-reported outcome–based symptom management [18], and patient symptom self-reporting systems [19]. Despite differences in terminology, these interventions share the common feature of enabling remote monitoring of patient symptoms through electronic platforms. To promote uniformity and standardization, this review adopts the term “remote digital symptom monitoring system (rSMS)” to refer to systems designed for digitally monitoring patient-reported outcomes.

The implementation of rSMS into routine cancer care for patients with lung cancer has gained attention in recent years, driven by its potential to enhance person-centered care [18,20]. However, evidence regarding its clinical impact remains inconsistent, reflecting heterogeneity in the evidence base, where benefits have been observed in certain domains but not consistently across all outcomes. While the efficacy of rSMS in alleviating symptom-related outcomes is widely reported, 1 randomized controlled trial (RCT) found no significant reductions in symptom burden among patients with lung cancer [21]. Furthermore, limitations in improving outcomes such as HRQL [22] and overall survival have also been documented among patients with lung cancer [17].

The integration of rSMS into lung cancer care has surged in recent years, yet the field remains marked by heterogeneity in system design, PROMs used, and clinical application. Studies vary widely in scope and sophistication: some prioritize basic functionalities, such as symptom data aggregation via mobile platforms to streamline communication between patients and health care providers [22,23], while other studies use advanced technologies to deliver real-time feedback to health care providers or patients, facilitating effective symptom management [17,24,25]. The most advanced systems integrate automated clinical alerts triggered by predefined symptom thresholds, potentially improving HRQL through timely interventions [26-28]. However, this diversity has resulted in fragmented implementation, with no unifying framework to categorize systems or evaluate their alignment with lung cancer–specific needs. It is important to note that theoretical frameworks play a critical role in enhancing the methodological rigor and reproducibility of rSMS intervention designs and implementations. Theory-driven interventions aimed at promoting behavior change are known to enhance intervention benefits [29-31].

To address these gaps, this scoping review focuses on the development and implementation of theory-driven rSMS interventions in lung cancer care. It aims to identify relevant studies and map how rSMS interventions have been designed, applied, and evaluated in this population, guided by the following questions: (1) “What theoretical frameworks have been applied to studies of rSMS in lung cancer?”; (2) “What are the most commonly used PROMs for rSMS in lung cancer?”; (3) “What are the key technology platforms, functionalities, and characteristics of rSMS?”; (4) “What are the experiences and perceptions of patients and health care providers regarding the implementation of rSMS?”; and (5) “What are the benefits and limitations of various rSMS interventions on clinical outcomes in patients with lung cancer?”

## Methods

### Review Design

This scoping review followed the Joanna Briggs Institute scoping review framework [32] and adhered to the PRISMA-ScR (Preferred Reporting Items for Systematic Reviews and Meta-Analyses Extension for Scoping Reviews) guidelines (Checklist 1) [33]. This protocol was registered on the Open Science Framework [34].

### Study Sources and Strategies

The literature search reporting adhered to the PRISMA-S (Preferred Reporting Items for Systematic Reviews and Meta-Analyses Literature Search Extension) guidelines (Checklist 2) [35]. Comprehensive literature searches were conducted from inception to April 3, 2025, in the following English and Chinese electronic databases: (1) PubMed, (2) CINAHL (via EBSCOhost), (3) Cochrane Library, (4) Embase, (5) Scopus, (6) Web of Science, (7) PsycINFO, (8) CNKI, (9) WanFang, and (10) SinoMed. The search strategy was developed collaboratively by the research team and peer-reviewed by an experienced librarian at the institution. Given the broad scope and objectives of this scoping review, the key search terms selected were “lung cancer,” “symptom monitoring,” “electronic patient-reported outcome,” and “remote monitoring.” Of note, no limits were applied for date, time period, search filters, or study design to maximize comprehensiveness. Both MeSH terms and free-text searches were used to ensure a thorough search. Additionally, the reference lists of included studies were reviewed to ensure no relevant evidence was overlooked [33]. If any included articles were found to contain potentially relevant information not provided in the original publication, the research team planned to obtain it by contacting the corresponding authors. The detailed search strategy used in each database is presented in Multimedia Appendix 1. The literature search was rerun on October 16, 2025, to update results.

### Eligibility Criteria

This study used the PCC (population, concept, and context) strategy to establish eligibility criteria in line with the Joanna Briggs Institute recommendations [32].

#### Population

Studies involving adults aged 18 years or older, diagnosed with any type of lung cancer (primary or secondary) across all disease stages (stages I-IV), and receiving any treatment modality, regardless of therapeutic intent (curative or palliative), were included. Studies involving mixed cancer groups were eligible if lung cancer–specific data—particularly regarding symptom severity—were extractable.

#### Concept

Studies focusing on the use of rSMS in lung cancer, where health-related outcomes are reported directly by patients through digital platforms.

#### Context

Studies examining the use of rSMS in patients with lung cancer, remotely from home or community-based settings.

### Types of Studies

This review included peer-reviewed primary studies (quantitative or qualitative) published in English or Chinese. Conference abstracts, reviews, comments, letters, and case reports were excluded, as these types of studies typically provide insufficient methodological detail or lack the ability to extract outcomes relevant to this review. Studies involving populations with mixed cancers, where lung cancer–specific data could not be isolated, were also excluded.

### Study Screening and Selection

All search results were exported to EndNote 2025 (Clarivate Analytics), where duplicates were removed by 2 reviewers independently (HH and TM). Titles and abstracts were thoroughly screened, followed by a full-text review to determine final inclusion or exclusion by 2 reviewers independently (HH and TM). Disagreements were discussed and resolved through consultation with 2 independent members of the team (NT and HA-O). The selection process is illustrated in a PRISMA-ScR flowchart [33].

### Data Extraction

Data relevant to the research questions were systematically extracted using a predesigned table developed by the research team. Specifically, the following details were extracted from each included study: (1) study characteristics (author, publication year, country, setting, sample size, and study design); (2) theoretical frameworks or models applied; (3) technology platforms for rSMS delivery and their key features (eg, accessibility, integration, and user interface); (4) clinical workflows for rSMS implementation (eg, with real-time clinician alerts and intervention, and automated responses to patients); (5) type of PROMs used and their symptom or health outcome domains; and (6) reported benefits and limitations of rSMS from patients, health care providers, and system perspectives.

For qualitative studies, thematic data on patients’ and health care providers’ experiences (eg, barriers, facilitators, and satisfaction) were also extracted.

### Data Synthesis

Quantitative data, including study characteristics, the use of PROMs, and reported benefits and barriers, were summarized in tabular and narrative formats, with a narrative interpretation provided. Qualitative data, such as user experiences, were analyzed using content analysis and synthesized narratively [36].

## Results

### Study Selection

**Figure 1.**
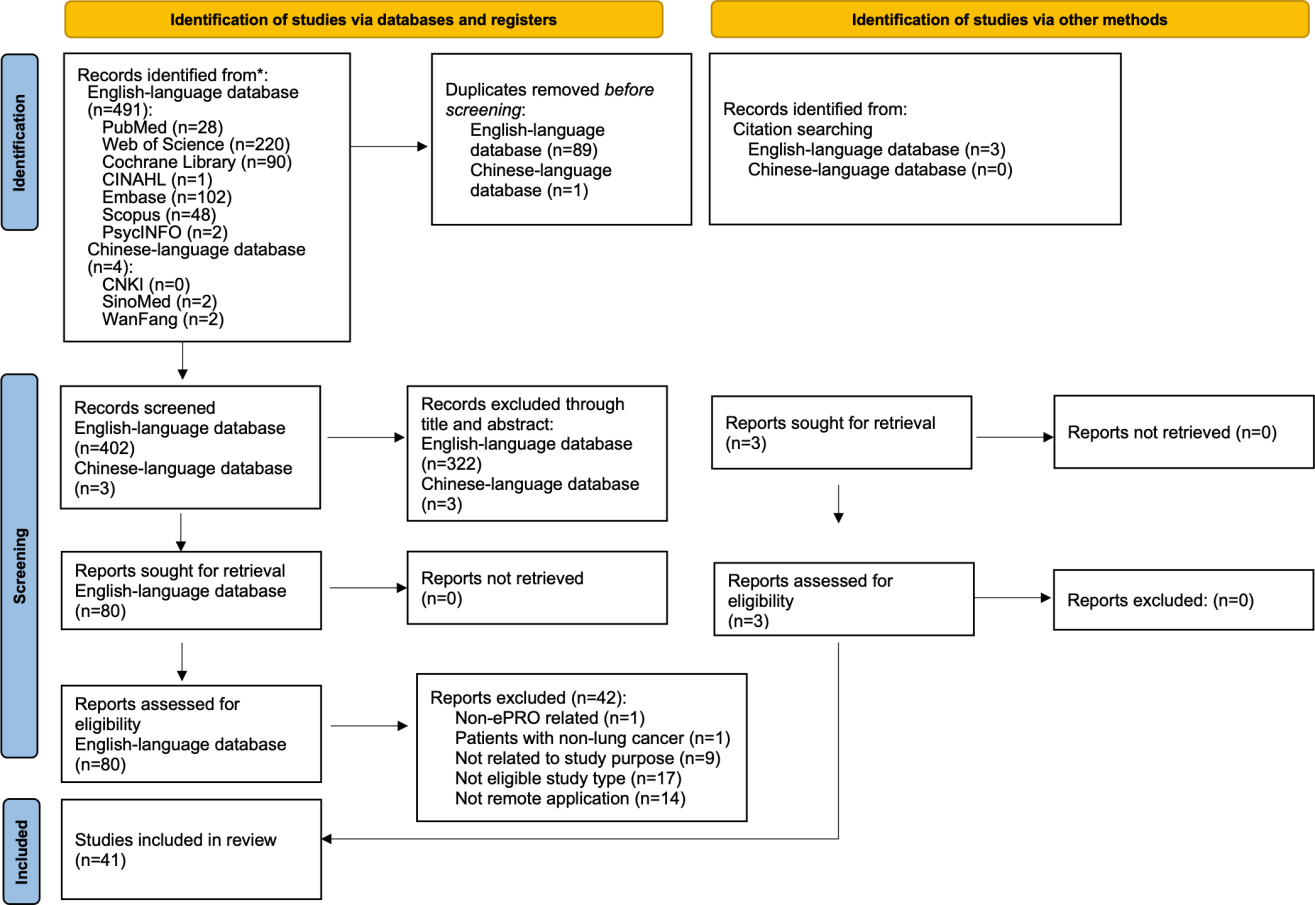
PRISMA (Preferred Reporting Items for Systematic Reviews and Meta-Analyses) flow diagram. ePRO: electronic patient-reported outcome.

A total of 495 articles were retrieved through the initial search, comprising 491 from English-language databases and 4 from Chinese-language databases. After screening titles and abstracts, followed by a thorough full-text review, 80 English-language articles were deemed eligible, while all Chinese-language articles were excluded due to irrelevant content or study designs that did not meet the inclusion criteria. Additionally, 3 studies were identified through citation searching. Ultimately, 41 studies were included in the final analysis. The search process is depicted in a PRISMA (Preferred Reporting Items for Systematic Reviews and Meta-Analyses) flowchart (Figure 1; Checklist 3).

### Study Characteristics

The 41 studies were published between 2007 and 2025. The majority of studies were conducted in the United States (n=19, 46%), followed by China (n=7, 17%), Denmark (n=3, 7%), the United Kingdom (n=3, 7%), and 1 (2%) study each from Australia, Germany, Spain, South Korea, Finland, France, Singapore, Italy, and Switzerland. The characteristics of the included studies are presented in Multimedia Appendix 2.

The majority of the included studies (n=19, 46%) used an RCT design, with 17 (41%) representing original RCTs [17,18,20-23,26,37-46] and 2 (5%) constituting secondary analyses derived from previously published RCTs [47,48]. The remaining publications encompassed prospective studies (n=8, 20%) [19,27,49-54], cohort studies (n=4, 10%) [55-57], mixed methods studies (n=4, 10%) [24,58-60], nonrandomized feasibility studies (n=4, 10%) [61-64], and qualitative studies (n=2, 5%) [65,66].

Among all the included studies, 5 (12%) enrolled health care providers as participants [49,50,58,60,63], whereas all others exclusively enrolled patients with lung cancer. Twelve (29%) studies focused exclusively on advanced-stage lung cancer [17,21,28,37,38,49,52,53,58,61,67], 7 (17%) included patients across all lung cancer stages [44,50,51,57,63,65,68], 3 (7%) targeted stages I to III [18,39,46], 1 (2%) concentrated on stages I to II [20], 1 (2%) focused on stage III [64], and 1 (2%) addressed stages II to IV [42]. Fifteen (37%) studies did not specify lung cancer stages [19,22-24,26,27,40,41,43,45,54-56,59,62].

Symptom monitoring across the included studies predominantly targeted respiratory complaints (cough, dyspnea, hemoptysis, voice changes, and chest pain), constitutional symptoms (fatigue, appetite loss, weight change, sleep disturbance, and cognitive difficulties), and gastrointestinal toxicities (nausea, vomiting, constipation, diarrhea, dysphagia, and sore mouth), as well as pain [17,18,20,21,39,42,46,51,55,61,62,64]. Psychological distress (anxiety, depression, and sadness) was less frequently assessed [21,42,45,46,55,64,67]. In addition, several research teams incorporated treatment-related adverse effects, such as side-effect bother, facial swelling, tumor growth sensation, and fever [17,42,45,61].

### Application of Theoretical Framework

Three studies noted the application of established theoretical frameworks to guide the rSMS development or intervention implementation (Table 1).

**Table 1.** Application of theoretical frameworks or guidelines in the development and implementation of a remote digital symptom monitoring system (rSMS) for lung cancer care.

Author (Year)	Framework or guideline	Purpose	Application
Maguire et al (2015) [59]	Holistic framework to improve the uptake and impact of eHealth technologies [69]	Development of the rSMS	Prospective
Girgis et al (2022) [24]	RE-AIM^a^ framework [70]	Guide the implementation	Retrospective
Mooney et al (2024) [26]	Chronic care model [71]	Development of the rSMS	Prospective

a RE-AIM: reach, effectiveness, adoption, implementation, and maintenance.

### Development of the rSMS

The application of theoretical foundations varied across studies. Maguire et al [59] applied the holistic framework to improve the uptake and impact of eHealth technologies and to guide the development of their rSMS [69]. This framework includes 6 key foundational components, namely stakeholder engagement, iterative evaluation, intertwined development and implementation, transformation of health care organizations, persuasive design techniques, and advanced methods for impact assessment. The chronic care model [71], a patient-centered framework that emphasizes proactive, planned, and systematic interventions, was prospectively applied to design the rSMS intervention and evaluation strategies by Mooney et al [26].

### Implementation of rSMS

Girgis et al [24] retrospectively applied the RE-AIM (reach, effectiveness, adoption, implementation, and maintenance) implementation science framework to guide and summarize successful intervention implementation, while concurrently identifying implementation barriers within this framework [70].

### PROMs Used Within the rSMS and/or as Study Outcome Measures

**Figure 2.**
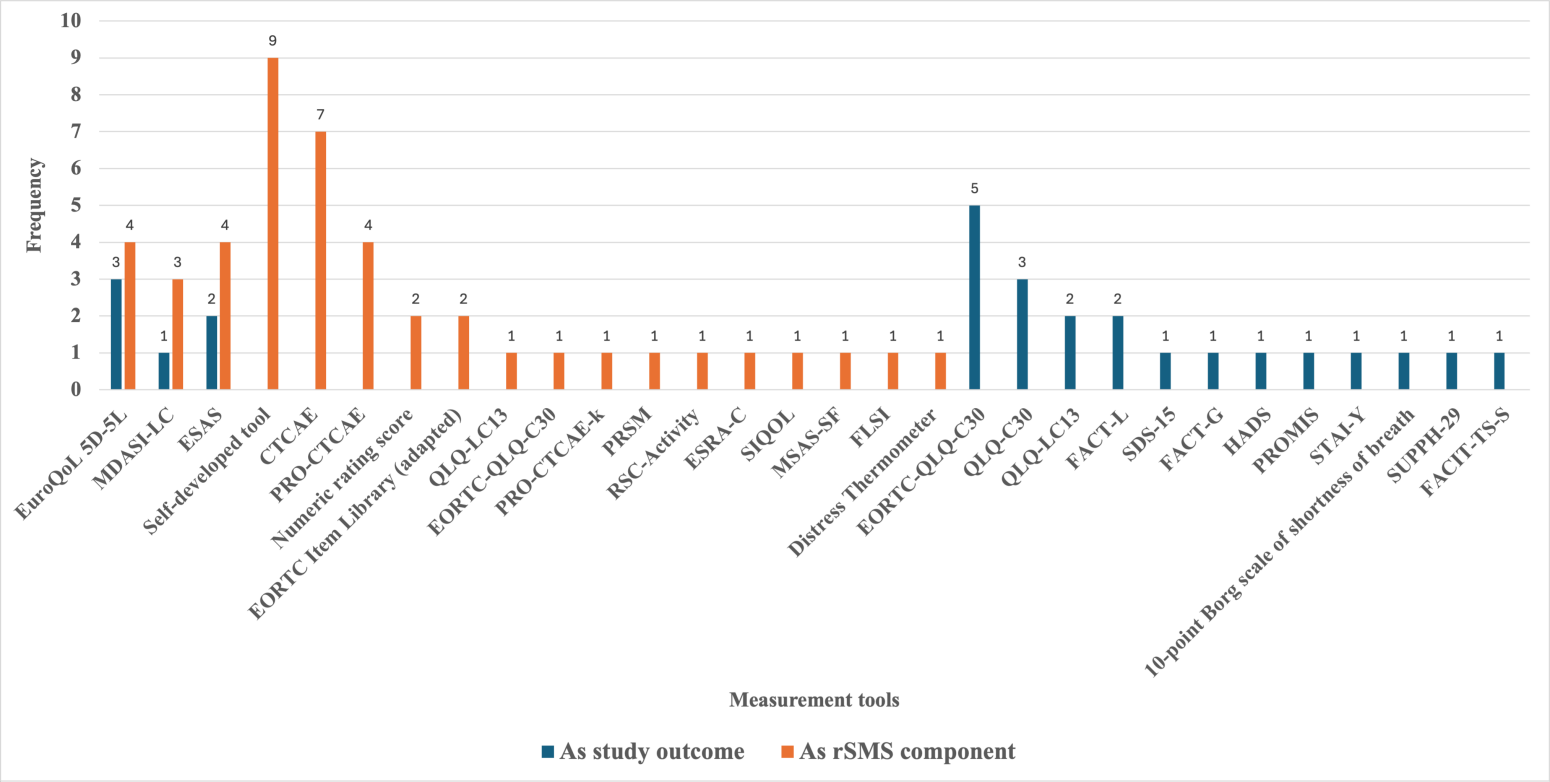
Frequency of patient-reported outcome measures applied as components in remote digital symptom monitoring systems (rSMS) and as outcome measures in studies on lung cancer symptom monitoring. CTCAE: Common Terminology Criteria for Adverse Events; EORTC-QLQ-C30: European Organisation for Research and Treatment of Cancer Core Quality of Life Questionnaire; ESAS: Edmonton Symptom Assessment Scale; ESRA-C: Electronic Self Report Assessment for Cancer; FACIT-TS-S: Functional Assessment of Chronic Illness Therapy-Treatment Satisfaction-Patient Satisfaction; FACT-G: Functional Assessment of Cancer Therapy–General; FACT-L: Functional Assessment of Cancer Therapy–Lung; FLSI: Functional Assessment of Cancer Therapy–Lung Symptom Index; HADS: Hospital Anxiety and Depression Scale; MDASI-LC: MD Anderson Symptom Inventory for Lung Cancer; MSAS-SF: Memorial Symptom Assessment Scale–Short Form; PRO-CTCAE: Patient-Reported Outcomes version of the Common Terminology Criteria for Adverse Events; PRO-CTCAE-k: Korean version of the Patient-Reported Outcomes version of the Common Terminology Criteria for Adverse Events; PROMIS: Patient-Reported Outcomes Measurement Information System; PRSM: Patient-Reported Symptom Monitoring; QLQ-LC13: European Organisation for Research and Treatment of Cancer Quality of Life Questionnaire Lung Cancer Module; RSC: Rotterdam Symptom Checklist–Activity Subscale; SDS-15: Symptom Distress Scale–15; SIQOL: Single Item Quality of Life scale; STAI-Y: State-Trait Anxiety Inventory–Form Y; SUPPH-29: Strategies Used by Patients to Promote Health–29.

A total of 32 PROMs applied across the included studies were categorized into two groups: (1) PROMs integrated into rSMS for monitoring purposes and (2) outcome measures used to evaluate the effectiveness of the rSMS intervention (Multimedia Appendix 3). Specifically, 19 PROMs were integrated within rSMS as components to monitor 3 domains—symptom assessment, HRQL, and psychological well-being. Thirteen PROMs were used as outcome measures to assess the effectiveness of the rSMS (Figure 2). Among all the 32 PROMs, three were used as both outcome measures and as components within the rSMS, including the Edmonton Symptom Assessment Scale, MD Anderson Symptom Inventory for Lung Cancer, and EuroQoL 5D-5L. Consistent with this review’s focus on rSMS development and implementation within lung cancer care, detailed analysis is restricted to PROMs integrated within the rSMS, while those functioning solely as outcome measures are comprehensively cataloged in Multimedia Appendix 4.

### Applied Technology Platforms and the Functional Modules: Technology Platforms

The included studies used a variety of platforms for rSMS, including web-based systems, mobile phones, and wearable devices. Most studies (n=21, 51%) used web-based systems for rSMS implementation [9,17,19,22,24,27,28,43,44,51,52,54,56-58,61,62,65-67]. Mobile phones were the second most used digital platform (n=14, 34%), facilitating real-time responses and notifications between health care providers and patients [20,21,23,26,37-41,45,46,55,59,60]. Six (15%) additional studies implemented rSMS through personal electronic devices—encompassing personal computers, smartphones, or other specialized devices [18,49,50,53,63,64].

### Functional Modules of the rSMS

**Figure 3.**
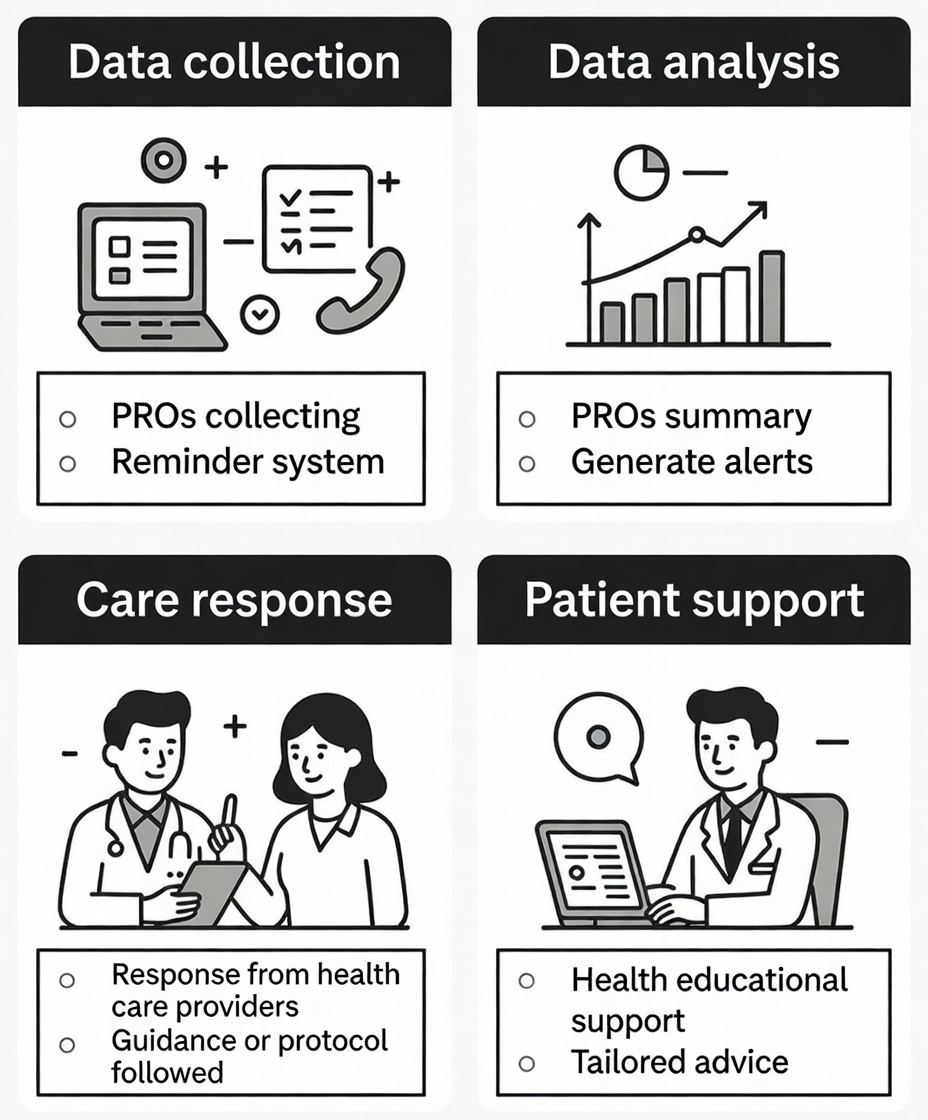
Functional modules of the remote digital symptom monitoring system. PRO: patient-reported outcome.

The functional modules of rSMS were categorized into 4 key stages: data collection, data analysis, care response, and patient support (Figure 3).

#### Data Collection

This stage involved symptom assessment tools to collect data, including symptom burden, symptom severity, HRQL, and other patient-reported outcomes. Additionally, 9 (22%) studies incorporated reminder systems to prompt timely patient assessments via emails, phone calls, or mobile app notifications [24,43,51,53,55,61,63,65,66].

#### Data Analysis

Following data collection, the data analysis stage was reported in all 41 studies. Of these, 10 (24%) studies reported systems generating summaries [22,23,38,40,42-44,60,62,72], which were provided to patients and/or health care providers, while others presented raw data or synthesized data manually. Additionally, 29 (71%) studies included notification or alert systems to inform the health care team when scores reached predefined thresholds [17-20,24,27,28,39-42,45,46,49,50,52-55,57-61,63-67]. Typically, this module automatically conducted data analysis and generated alerts when predefined clinical criteria were met, which were then transmitted to the clinical teams—a mechanism that has been effectively implemented in current practice [17,42,49].

#### Care Response

The clinical response stage required mandatory responses from health care providers within a predefined time window, as reported in 11 (27%) studies [18,26,28,37,39,44-46,55,57,63]. A representative implementation by Dai et al’s [18] study stipulated that when the rSMS identified a patient-reported symptom score that exceeded 4 points (indicating moderate-to-severe severity), surgeons who were involved in the patient’s care were obligated to respond within 24 hours in accordance with the study protocol.

#### Patient Support

There were 16 (39%) studies that provided additional health education support based on the varying levels of symptom burden reported by patients [20,23,24,26,27,37,40,41,44,46,49,54,58,59,63,64]. For instance, the MyChristie-MyHealth program categorizes patients using a color-coded system based on symptom severity and provides tailored support advice [50]. Asymptomatic (“green”) patients received a text message indicating that no action was required, mild symptoms (“blue”) triggered an automated link to self-care advice, moderate symptoms (“orange”) prompted patients to seek support from their oncology team within 1 week, and patients with severe symptoms (“red”) received a message recommending urgent medical advice within 24 hours.

### Experiences and Perceptions of Patients and Health Care Professionals

**Figure 4.**
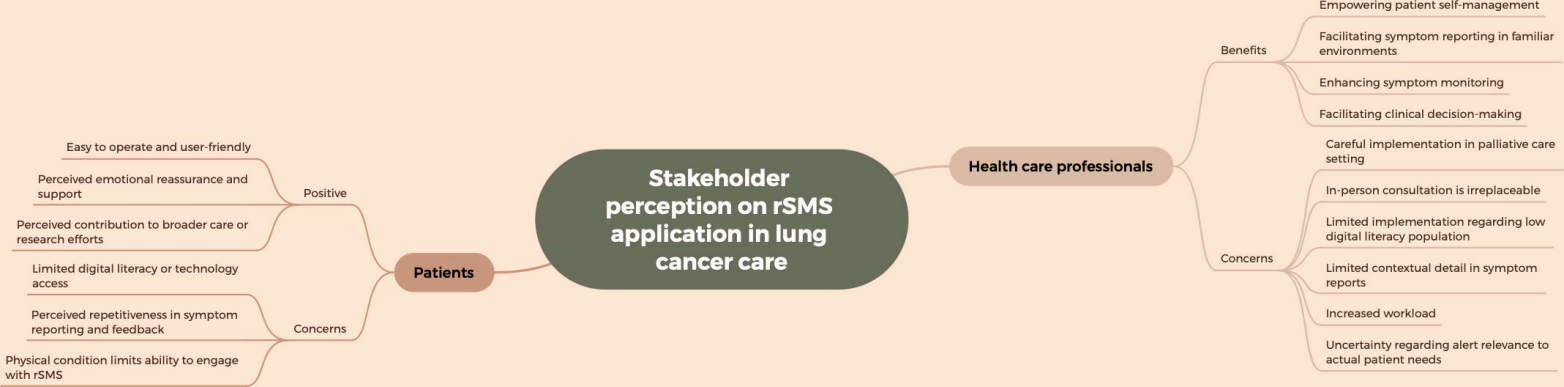
Perceived benefits and concerns of the remote symptom monitoring system (rSMS) application among patients and health care professionals.

User experiences of both patients and health care providers were evaluated through semistructured interviews across 5 (12%) studies [45,59,60,63,66]. Questionnaire-based surveys were also used to measure satisfaction levels among patients and health care providers [62,63]. Additionally, qualitative interviews were conducted to examine the feasibility of interventions and to identify facilitating and hindering factors from the patients’ perspective [65]. The findings from the content analysis are detailed in Multimedia Appendix 5 and visually synthesized in Figure 4, which highlights the key benefits and concerns expressed by patients and health care professionals regarding rSMS use.

### Perceptions of rSMS Applications Among Health Care Professionals

Health care providers expressed diverse benefits and barriers of rSMS. First, some health care providers in palliative care settings perceived that proactive monitoring could empower certain patients by granting them greater control over their conditions and enhancing their disease management abilities, as the system provided positive feedback that motivated patients and guided their next steps [60]. However, the same study included no patient outcome data, and most health care providers expressed doubts about implementing such interventions in palliative care settings. This aligns with the concerns voiced by clinicians in other research [63]. Additionally, the remote reporting feature allowed patients to report symptoms in familiar surroundings, potentially fostering a more relaxed reporting experience compared to hospital settings [66]. Second, for health care providers, patients’ previsit self-reports enabled the care teams to prepare more effectively and facilitated early identification of symptom changes [59]. Regular monitoring of symptom fluctuations also supported disease progression tracking, aiding timely adjustments to care strategies and clinical decision-making [63,66].

Concerns about rSMS implementation were also reported. Some physicians emphasized that rSMS should supplement rather than replace clinical judgment [60,66]. Similarly, other clinicians highlighted that traditional in-person consultations and hands-on experience should not be supplanted by remote reporting, as reliance solely on patient self-reports without clinical observation poses significant challenges [60,66]. Additional concerns included the selective applicability of rSMS, particularly regarding older patients’ unfamiliarity with or resistance to electronic systems, as well as its limited benefits for palliative care groups [60]. Regarding rSMS itself, some health care providers identified limitations in its ability to support rigorous clinical decisions, noting that symptom reports often lack contextual details such as frequency or type, necessitating further patient interaction [66]. Additionally, some health care providers reported that rSMS added to their existing workload, making timely responses challenging [59,66]. Furthermore, health care providers expressed concerns about alerts, questioning whether all alerts are necessary and effectively reflect patients’ actual symptom burdens, while also worrying that excessive alerts might increase workloads [63].

### Perceptions of rSMS Applications Among Patients

Patients reported that rSMS was generally user-friendly and easy to operate, enabling them to conveniently report their symptoms and functional status [59,65]. Timely response systems and tailored health advice were considered highly helpful [45,59,65]. Some patients felt that rSMS provided a sense of safety, encouragement, and support, making them feel attended to by health care providers outside clinical settings [45,59,65]. Additionally, patients reported feeling that they were not only receiving help during the intervention, but also contributing to helping others [45].

However, some patients expressed concerns about rSMS. System-related barriers included hardware issues (eg, unstable access in rural areas), a preference for paper-based surveys, and unfamiliarity with electronic systems [9,65]. Some perceived symptom assessments and advice as redundant with existing knowledge, reducing motivation to complete surveys [59,65]. Others questioned rSMS efficacy, describing it as “self-limited” [59,65]. High symptom burden was also cited as an engagement barrier [65].

### Clinical Insights

**Figure 5.**
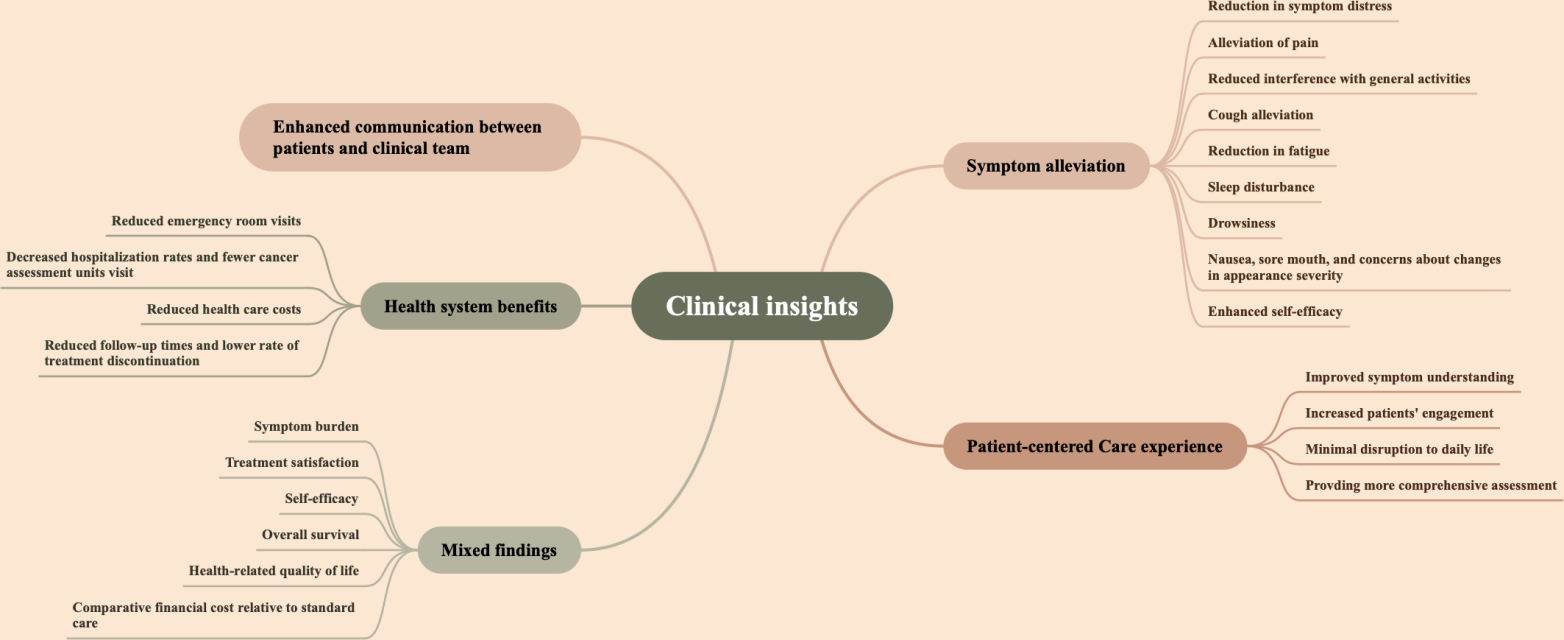
Key clinical domains impacted by remote symptom monitoring systems (rSMS).

The implementation of rSMS among patients with lung cancer has demonstrated substantial benefits, including symptom alleviation, improvements in HRQL, enhanced communication between patients and health care professionals, strengthened patient-centered care experiences, and broader health system advantages. Nevertheless, some studies have reported nonsignificant effects on clinical outcomes, underscoring the variability of evidence (Figure 5).

#### Symptom-Related Outcome

The rSMS intervention demonstrated significant reductions in overall symptoms among patients with lung cancer [18,20,22,26,39,41,46,52]. Symptom distress emerged as the most frequently documented alleviation and was evident across 4 (10%) studies [22,41,46,59]. This was followed by a significant decrease in patient-reported pain, noted in 2 RCTs [20,41]. Additionally, rSMS was reported to notably reduce interference with general activities, work, and walking, as observed in 2 RCTs [18,20]. Significant clinical improvements were reported in cough [20], fatigue [20,41], sleep disturbance [20,41], and drowsiness [59]. One (2%) study extended these findings to additional symptom categories—including nausea, sore mouth, and concerns about changes in appearance severity [41]. Beyond physical symptom management, 1 (2%) study observed enhanced patient self-efficacy, attributing these gains to rSMS-facilitated psychological distress reduction and improved adaptive functioning [59]. Conversely, another research team reported no significant improvements in symptom burden, treatment satisfaction, or self-efficacy [21]. Similarly, no significant rSMS-related benefits in overall survival were found in a separate study [17].

#### Health-Related Quality of Life

Five (12%) studies observed improvements in HRQL among patients using a symptom monitoring system [17,40,42,43,46]. However, 4 (10%) studies revealed no statistically significant improvements in HRQL among rSMS intervention groups [21,22,37,63].

#### Communication With Health Care Providers

Five (12%) studies reported that the rSMS application enhanced communication or improved discussions between patients and health care providers [22,49,50,52,57]. All these studies used questionnaire-based investigations to assess patients’ perceptions. Additionally, a pooled analysis suggested that improved communication may constitute one of the potential reasons for the success of the intervention program [67]. For health care providers, rSMS primarily supported clinical workflows and optimized patient communication. This includes reducing time spent on follow-up tracking [39], enhancing work efficiency [39,40,58], and increasing confidence [24] in symptom monitoring and management.

#### Patient-Centered Care Experience

Several studies have demonstrated that rSMS contributes to modern patient-centered care through various approaches. Three (7%) studies reported improved symptom understanding using a patient-reported experience measure in rSMS for lung cancer care [44,49,50]. Additionally, this intervention enhanced patient-care team interactions, increasing patients’ engagement in their own care [50]. Furthermore, 2 RCTs reported that rSMS functioned effectively without disrupting daily lives, yielding high patient satisfaction in lung cancer care [19,39]. Similarly, rSMS enhanced patients’ perceived benefit, making them feel their cancer care significantly improved [19,53]. Implementation also provided significant clinical benefits for specific lung cancer groups, such as patients receiving immune checkpoint inhibitors, who were reported to have a more comprehensive assessment, including toxicity grading and long-term surveillance of symptoms [40].

#### Health System Benefits

Reduced emergency room visits were reported in 2 (5%) studies [40,43]. Similarly, decreased hospitalization rates and fewer visits to cancer assessment units were reported in 2 (5%) other studies [56,73]. Patt et al further identified that rSMS was associated with a lower total cost of care [56]. One RCT study reported that enhanced treatment experiences and automated platforms contributed to reduced follow-up times and a lower rate of treatment discontinuation [40]. Additionally, financial costs were compared between rSMS implementation and the standard of care, with no statistically significant differences observed [45]. In the PROStep feasibility study [38], which integrated both self-reported surveys and passive activity monitoring for patients with lung and gastrointestinal cancers—whereas most other platforms in our review relied on active reporting only—the intervention group reported a high perceived understanding of symptoms and functional status by their oncology team; this did not differ from the control group [38].

## Discussion

### Principal Findings

This scoping review provides an overview of how rSMS has been applied in lung cancer care, with particular attention to the integration of PROMs and functional modules. The synthesis of existing studies indicates that rSMS interventions show encouraging potential to improve symptom surveillance and support timely clinical responses; however, the current evidence remains fragmented and methodologically limited. Notably, few studies have adopted theory-driven approaches in the design, implementation, or evaluation of rSMS, leaving important conceptual and practical gaps. In addition, variability in patient and health care provider engagement highlights ongoing challenges related to usability and comprehension. These findings set the stage for a more detailed discussion of the opportunities and challenges in advancing rSMS, as well as directions for future research.

Only 3 (7%) studies incorporated theory-driven approaches in designing the intervention program or evaluating the outcomes [24,26,59]. Existing evidence from implementation science underscores that theory-driven approaches enhance methodological transparency, clarify intervention mechanisms, and promote sustainability [74,75]. The current limited application of theoretical frameworks may contribute to inconsistencies in intervention implementation and hinder the comparability and validity of outcome measurements across studies. Future studies should integrate theoretical guidance at both the design and evaluation stages to maximize the effectiveness and sustainability of interventions.

To ensure the reliability of patient-reported data and the validity of study outcomes, rSMS should incorporate PROMs that have undergone rigorous psychometric validation. However, as illustrated in Figure 2, the majority of PROMs used in rSMS studies were self-developed by research teams. Of the 9 self-developed tools identified, only 4 (10%) studies explicitly reported having conducted formal validation procedures [26,42,55]. The limited reporting of validation efforts may affect the interpretability and generalizability of findings derived from these tools, underscoring the need for greater emphasis on validated PROMs in future rSMS designs.

This review further delineates four key functional modules characterizing current rSMS applications in lung cancer care, including data collection, data analysis, the response system, and patient support. While most platforms offer real-time alert capabilities, more than half report limited clinical action in response to these alerts. This lack of action is primarily attributed to barriers such as competing clinical demands and unclear delineation of responsibilities [66]. This highlights a critical need for integration strategies that link real-time symptom alerts to actionable care pathways and effective feedback mechanisms, ensuring digital monitoring enables timely intervention rather than functioning as a passive data repository.

The qualitative findings identified in this review remain limited, with reported outcomes demonstrating variability [45,59,60,63,65,66]. While health care providers value rSMS for symptom monitoring, they noted limitations, including reduced effectiveness among patients with limited digital literacy—particularly older patients with lung cancer—and perceptions of data subjectivity, possibly due to misunderstandings about the system [60,66]. Patients acknowledge rSMS benefits but express notable concerns regarding the rSMS system’s trustworthiness [65]. A deeper understanding of these perceptions and experiences is needed to inform strategies for patient engagement and health care provider integration. Importantly, current evidence predominantly originates from Western high-income settings with advanced medical infrastructure, highlighting the need for research in diverse resource environments to comprehensively understand rSMS implementation.

Clinical outcomes associated with rSMS interventions were found to be heterogeneous. Several trials reported meaningful benefits, including reductions in symptom distress and improvements in pain, fatigue, or sleep disturbance [18,20,22,26,39,41,46,52]. In contrast, other studies observed limited or no impact on broader end points such as global quality of life, overall survival, or treatment satisfaction [21,22,37,63]. These discrepancies may be explained by heterogeneity in rSMS design and implementation, including differences in clinical context, monitoring frequency, patient reporting requirements, and the extent of clinician engagement. Context-related factors may also contribute to this variability, with effectiveness potentially differing across specific care settings. For example, prior work has highlighted that the use of rSMS in palliative care requires particular caution to avoid imposing additional burdens on patients receiving end-of-life care [60].

### Future Directions

In the era of precision medicine, rSMS integration can be optimized through person-centered interventions tailored to the diverse disease trajectories of patients with lung cancer. Such a tailored approach could enhance symptom assessment and management efficacy. For example, identifying variations in symptom burden can enable the integration of personalized education and technical support into the intervention program. The color-coded system identified in this review exemplifies a tailored tool for assessing these variations, thereby optimizing the application of rSMS [50].

Improving patients’ understanding of rSMS implementation could facilitate its successful adoption. While the current exploratory evidence is limited, studies indicate that patients frequently hesitate to seek medical assistance despite receiving alerts [49,50]. The observed reluctance to seek medical help may reflect a limited comprehension of the intervention’s objectives and functionality. As patients’ understanding of rSMS can influence their adherence and engagement, future studies should prioritize preintervention education on rSMS features and clinical aims to reinforce engagement.

### Limitations

This review has several limitations. First, the included studies were predominantly published in English. Although Chinese literature underwent initial screening, no studies met the inclusion criteria. Second, consistent with the guidelines of scoping review methodology, no formal quality appraisal of the included studies was performed, and therefore, the methodological quality of the evidence could not be ascertained. Third, due to the considerable heterogeneity in study designs, interventions, and outcome measures, it was challenging to draw definitive conclusions regarding clinical effectiveness.

### Conclusions

This review advances the field by offering a comprehensive synthesis of the landscape of rSMS in lung cancer care. Unlike previous reviews that focused primarily on clinical outcomes or technical feasibility, this review adds to prior work by summarizing the theoretical foundations guiding rSMS, integrating functional modules, and distinguishing PROMs used as embedded system components from those reported as outcome measures. Together, these contributions clarify the current landscape and highlight methodological gaps. This is innovative because it provides a theoretical lens for comparing rSMS, not only their outcomes. To ensure long-term sustainability and clinical relevance, future research should adopt theory-driven approaches, refine functional integration, and incorporate multistakeholder perspectives to develop rSMS platforms that are patient-centered, sustainable, and adaptable across diverse clinical contexts while minimizing clinician burden.

## Supplementary material

10.2196/83666 Multimedia Appendix 1 Overview of search strategy used across all databases.

10.2196/83666 Multimedia Appendix 2 Characteristics of included studies.

10.2196/83666 Multimedia Appendix 3 Patient-reported outcome measures serve as both components and outcome measures within remote digital symptom monitoring system.

10.2196/83666 Multimedia Appendix 4 Patient-reported outcome measures serve as outcome indicators.

10.2196/83666 Multimedia Appendix 5 Content analysis of qualitative studies.

10.2196/83666 Checklist 1 PRISMA-ScR checklist

10.2196/83666 Checklist 2 PRISMA-S checklist

10.2196/83666 Checklist 3 PRISMA checklist

## Abbreviations

ePROM: electronic patient-reported outcome measure
HRQL: health-related quality of life
PCC: population, concept, and context
PRISMA: Preferred Reporting Items for Systematic Reviews and Meta-Analyses
PRISMA-S: Preferred Reporting Items for Systematic Reviews and Meta-Analyses Extension for Literature Search
PRISMA-ScR: Preferred Reporting Items for Systematic Reviews and Meta-Analysis Extension for Scoping Reviews
PROM: patient-reported outcome measure
RCT: randomized controlled trial
RE-AIM: reach, effectiveness, adoption, implementation, and maintenance
rSMS: remote digital symptom monitoring system
