# Supplementary material for: The Current Landscape of Remote Digital Symptom Monitoring for Patients With Lung Cancer: Scoping Review
Source: J Med Internet Res. 2026 Mar 24;28:e83666. doi: 10.2196/83666 (PMC13012230; doi:10.2196/83666)
Supplement: Multimedia Appendix 1 [file jmir-v28-e83666-s001.docx]

**Appendix 1 Search Strategy**

PubMed

| Search | Query |
| --- | --- |
| #1 | lung neoplasms[MeSH Terms] |
| #2 | ((((lung cancer[Title/Abstract]) OR (lung neoplasm*[Title/Abstract])) OR (lung carcinoma[Title/Abstract])) OR (pulmonary neoplasm*[Title/Abstract])) OR (pulmonary cancer[Title/Abstract]) |
| #3 | #1 OR #2 |
| #4 | Patient Reported Outcome Measures[MESH Terms] |
| #5 | ((((Patient Reported Outcome Measure[Title/Abstract]) OR (Patient Reported Outcomes[Title/Abstract])) OR (Patient-Reported Outcome[Title/Abstract])) OR (Patient-Reported Outcomes[Title/Abstract])) OR (Patient Reported Outcome[Title/Abstract]) |
| #6 | #4 OR #5 |
| #7 | ((Internet-Based Intervention[MeSH Terms]) OR (telemedicine[MeSH Terms])) OR (telerehabilitation[MeSH Terms]) |
| #8 | (((((((((((((('web'[Title/Abstract]) OR ('internet'[Title/Abstract])) OR ('internet based'[Title/Abstract])) OR ('web based'[Title/Abstract])) OR ('telecommunication*'[Title/Abstract])) OR ('mobile technology'[Title/Abstract])) OR ('mhealth'[Title/Abstract])) OR ('mcare'[Title/Abstract])) OR ('electronic patient reported outcome*'[Title/Abstract])) OR ('electronic patient-reported outcome*'[Title/Abstract])) OR ('epro*'[Title/Abstract])) OR ('remote digital'[Title/Abstract])) OR ('digital'[Title/Abstract])) OR ('remote monitor*'[Title/Abstract])) OR ('remote'[Title/Abstract]) |
| #9 | #7 OR #8 |
| #10 | #6 AND #9 |
| #11 | Symptom assessment[Mesh Terms] |
| #12 | (((symptom management[Title/Abstract]) OR (symptom control[Title/Abstract])) OR (symptom assessment[Title/Abstract])) OR (symptom monitor*[Title/Abstract]) |
| #13 | #11 OR #12 |
| #14 | #3 AND #10 AND #13 |

CINAHL (via EBSCOhost)

| Search | Query |
| --- | --- |
| S1 | ((MM "Patient Assessment+") OR (SU symptom management or symptom control or remission or relief or improvement)) AND (((MM "Patient-Reported Outcomes+") OR (SU 'patient reported outcome measure' OR SU 'patient reported outcomes' OR SU 'patient-reported outcome' OR SU 'patient-reported outcomes' OR SU 'patient reported outcome')) |
| S2 | ((MM "Internet-Based Intervention" OR MM "Telemedicine+" OR MM "Telerehabilitation") OR (SU 'web' OR SU 'internet' OR SU 'internet based' OR SU 'web based' OR SU 'telecommunication*' OR SU 'mobile technology' OR SU ('mhealth' OR 'mcare' OR 'electronic patient reported outcome*' OR 'electronic patient-reported outcome*' OR 'epro*' OR 'remote digital' OR 'digital' OR 'remote monitor*' OR 'remote')))) |
| S3 | ((SU 'lung tumor' OR SU 'lung cancer' OR SU 'lung neoplasm*' OR SU 'lung carcinoma' OR SU 'pulmonary neoplasm*' OR SU 'pulmonary cancer') OR (MM Lung Neoplasms)) |
| S4 | S1 AND S2 AND S3 |

Cochrane Library

| Search | Query |
| --- | --- |
| #1 | ('lung tumor'):ti,ab,kw OR ('lung cancer'):ti,ab,kw OR ('lung neoplasm*'):ti,ab,kw OR ('lung carcinoma'):ti,ab,kw OR ('pulmonary neoplasm*' OR 'pulmonary cancer'):ti,ab,kw |
| #2 | MeSH descriptor: [Lung Neoplasms] explode all trees |
| #3 | #1 OR #2 |
| #4 | MeSH descriptor: [Patient Reported Outcome Measures] explode all trees |
| #5 | ('patient reported outcome measure'):ti,ab,kw OR ('patient reported outcomes'):ti,ab,kw OR ('patient-reported outcome'):ti,ab,kw OR ('patient-reported outcomes'):ti,ab,kw OR ('patient reported outcome'):ti,ab,kw |
| #6 | ('web' OR 'internet' OR 'internet based' OR 'web based' OR 'telecommunication*' OR 'mobile technology'):ti,ab,kw OR ('mhealth' OR 'mcare' OR 'electronic patient reported outcome*' OR 'electronic patient-reported outcome*'):ti,ab,kw OR ('epro*' OR 'remote digital'):ti,ab,kw OR ('digital' OR 'remote monitor*'):ti,ab,kw OR ('remote'):ti,ab,kw |
| #7 | MeSH descriptor: [Internet-Based Intervention] explode all trees |
| #8 | MeSH descriptor: [Telemedicine] explode all trees |
| #9 | MeSH descriptor: [Telerehabilitation] explode all trees |
| #10 | #6 OR #7 OR #8 OR #9 |
| #11 | #4 OR #5 |
| #12 | MeSH descriptor: [Symptom Assessment] explode all trees |
| #13 | ('symptom management'):ti,ab,kw OR ('symptom control'):ti,ab,kw OR ('symptom assessment'):ti,ab,kw OR ('symptom monitor*'):ti,ab,kw |
| #14 | #12 OR #13 |
| #15 | #10 AND #11 |
| #16 | #3 AND #14 AND #15 |

Embase

| Search | Query |
| --- | --- |
| #1 | 'lung tumor'/exp OR 'lung cancer':ti,ab,kw OR 'lung neoplasm*':ti,ab,kw OR 'lung carcinoma':ti,ab,kw OR 'pulmonary neoplasm*':ti,ab,kw OR 'pulmonary cancer':ti,ab,kw |
| #2 | 'patient-reported outcome'/exp OR 'patient reported outcome measure':ti,ab,kw OR 'patient reported outcomes':ti,ab,kw OR 'patient-reported outcome':ti,ab,kw OR 'patient-reported outcomes':ti,ab,kw OR 'patient reported outcome':ti,ab,kw |
| #3 | 'web':ti,ab,kw OR 'internet':ti,ab,kw OR 'internet based':ti,ab,kw OR 'web based':ti,ab,kw OR telecommunication*:ti,ab,kw OR 'mobile technology':ti,ab,kw OR 'mhealth':ti,ab,kw OR 'mcare':ti,ab,kw OR 'electronic patient reported outcome*':ti,ab,kw OR 'electronic patient-reported outcome*':ti,ab,kw OR 'epro*':ti,ab,kw OR 'digital':ti,ab,kw OR 'remote':ti,ab,kw OR 'remote digital':ti,ab,kw OR 'remote monitor*':ti,ab,kw |
| #4 | 'web-based intervention'/exp OR 'telemedicine'/exp OR 'telerehabilitation'/exp |
| #5 | #3 OR #4 |
| #6 | #2 AND #5 |
| #7 | 'symptom assessment'/exp OR 'symptom management':ti,ab,kw OR 'symptom control':ti,ab,kw OR 'symptom assessment':ti,ab,kw OR 'symptom monitor*':ti,ab,kw |
| #8 | #1 AND #6 AND #7 |

Scopus

| Search | Query |
| --- | --- |
| #1 | TITLE-ABS-KEY ("lung cancer" OR "lung neoplasm*" OR "lung carcinoma" OR "pulmonary neoplasm*" OR "pulmonary cancer") |
| #2 | (TITLE-ABS-KEY ("patient reported outcome measures") OR TITLE-ABS-KEY ("patient reported outcomes") OR TITLE-ABS-KEY ("patient-reported outcome") OR TITLE-ABS-KEY ("patient-reported outcomes") OR TITLE-ABS-KEY ("patient reported outcome") ) |
| #3 | (TITLE-ABS-KEY ("internet-based intervention") OR TITLE-ABS-KEY ("telemedicine") OR TITLE-ABS-KEY ("telerehabilitation") OR TITLE-ABS-KEY ("web") OR TITLE-ABS-KEY ( "internet") OR TITLE-ABS-KEY ("internet based" ) OR TITLE-ABS-KEY ( "web-based") OR TITLE-ABS-KEY ( "web based") OR TITLE-ABS-KEY ( "telecommunication*") OR TITLE-ABS-KEY ("mobile technology") OR TITLE-ABS-KEY ("mhealth" ) OR TITLE-ABS-KEY ("e-health") OR TITLE-ABS-KEY ("m-health") OR TITLE-ABS-KEY ("m-care" ) OR TITLE-ABS-KEY ( "electronic patient reported outcome*") OR TITLE-ABS-KEY ("electronic patient-reported outcome*") OR TITLE-ABS-KEY ("epro*") )\| |
| #4 | ( ( TITLE-ABS-KEY ("internet-based intervention") OR TITLE-ABS-KEY ("telemedicine") OR TITLE-ABS-KEY ("telerehabilitation" ) OR TITLE-ABS-KEY ("web" ) OR TITLE-ABS-KEY ("internet") OR TITLE-ABS-KEY ("internet based" ) OR TITLE-ABS-KEY ( "web-based" ) OR TITLE-ABS-KEY ( "web based") OR TITLE-ABS-KEY ("telecommunication*") OR TITLE-ABS-KEY ("mobile technology") OR TITLE-ABS-KEY ("mhealth" ) OR TITLE-ABS-KEY ("e-health") OR TITLE-ABS-KEY ("m-health") OR TITLE-ABS-KEY ("m-care") OR TITLE-ABS-KEY ("electronic patient reported outcome*") OR TITLE-ABS-KEY ("electronic patient-reported outcome*") OR TITLE-ABS-KEY ("epro*"))) AND ((TITLE-ABS-KEY ("patient reported outcome measures") OR TITLE-ABS-KEY ( "patient reported outcomes") OR TITLE-ABS-KEY ("patient-reported outcome") OR TITLE-ABS-KEY ( "patient-reported outcomes") OR TITLE-ABS-KEY ("patient reported outcome")) )\| |
| #5 | (TITLE-ABS-KEY ("symptom assessment") OR TITLE-ABS-KEY ("symptom management") OR TITLE-ABS-KEY ("symptom control") OR TITLE-ABS-KEY ("symptom monitor*") ) |
| #6 | (TITLE-ABS-KEY ("lung cancer" OR "lung neoplasm*" OR "lung carcinoma" OR "pulmonary neoplasm*" OR "pulmonary cancer")) AND (( (TITLE-ABS-KEY ("internet-based intervention") OR TITLE-ABS-KEY ( "telemedicine") OR TITLE-ABS-KEY ("telerehabilitation") OR TITLE-ABS-KEY ("web" ) OR TITLE-ABS-KEY ("internet") OR TITLE-ABS-KEY ("internet based") OR TITLE-ABS-KEY ( "web-based" ) OR TITLE-ABS-KEY ( "web based" ) OR TITLE-ABS-KEY ("telecommunication*") OR TITLE-ABS-KEY ("mobile technology") OR TITLE-ABS-KEY ("mhealth") OR TITLE-ABS-KEY ("e-health" ) OR TITLE-ABS-KEY ("m-health") OR TITLE-ABS-KEY ("m-care") OR TITLE-ABS-KEY ("electronic patient reported outcome*") OR TITLE-ABS-KEY ("electronic patient-reported outcome*") OR TITLE-ABS-KEY ("epro*"))) AND ( (TITLE-ABS-KEY ("patient reported outcome measures") OR TITLE-ABS-KEY ("patient reported outcomes") OR TITLE-ABS-KEY ( "patient-reported outcome") OR TITLE-ABS-KEY ("patient-reported outcomes") OR TITLE-ABS-KEY ( "patient reported outcome" )) AND ((TITLE-ABS-KEY ("symptom assessment") OR TITLE-ABS-KEY ("symptom management") OR TITLE-ABS-KEY ("symptom control") OR TITLE-ABS-KEY ("symptom monitor*")) ) |

Web of Science

| Search | Query |
| --- | --- |
| #1 | ((((TS=('symptom assessment')) OR TS=('symptom management')) OR TS=('symptom control')) OR TS=('symptom assessment')) OR TS=('symptom monitor*') |
| #2 | (((((TS=('patient-reported outcome')) OR TS=('patient reported outcome measure')) OR TS=('patient reported outcomes')) OR TS=('patient-reported outcome')) OR TS=('patient-reported outcomes')) OR TS=('patient reported outcome') |
| #3 | (((((((((((((((((TS=('web')) OR TS=('internet')) OR TS=('internet based')) OR TS=('web based')) OR TS=('telecommunication*')) OR TS=('mobile technology')) OR TS=('mhealth')) OR TS=('mcare')) OR TS=('electronic patient reported outcome*')) OR TS=('electronic patient-reported outcome*')) OR TS=('epro*')) OR TS=('digital')) OR TS=('remote')) OR TS=('remote digital')) OR TS=('remote monitor*')) OR TS=('web-based intervention')) OR TS=('telemedicine')) OR TS=('telerehabilitation') |
| #4 | #2 AND #3 |
| #5 | (((((TS=('lung tumor')) OR TS=('lung cancer')) OR TS=('lung neoplasm*')) OR TS=('lung carcinoma')) OR TS=('pulmonary neoplasm*')) OR TS=('pulmonary cancer') |
| #6 | #1 AND #4 AND #5 |

PsycINFO

| Search | Query |
| --- | --- |
| S1 | mainsubject("lung neoplasms") OR summary("lung cancer") OR summary("lung neoplasm*") OR summary("lung carcinoma") OR summary("pulmonary neoplasm*") OR summary("pulmonary cancer") |
| S2 | mainsubject("Patient Reported Outcome Measures") OR summary("Patient Reported Outcome Measure") OR summary("Patient Reported Outcomes") OR summary("Patient-Reported Outcomes") OR summary("Patient-Reported Outcome") OR summary("Patient Reported Outcome") |
| S3 | (mainsubject("Internet-Based Intervention") OR mainsubject("telemedicine") OR mainsubject("telerehabilitation")) |
| S4 | (summary("web") OR summary("internet") OR summary("internet based") OR summary("web-based") OR summary("web based") OR summary("telecommunication") OR summary("telecommunications") OR summary("mobile technology") OR summary("mhealth") OR summary("e-health")) |
| S5 | (summary("m-health") OR summary("m-care") OR summary("electronic patient reported outcome*") OR summary("electronic patient-reported outcome*)) |
| S6 | [S3] OR [S4] OR [S5] |
| S7 | [S2] AND [S6] |
| S8 | (mainsubject("Symptom assessment") OR summary("symptom management") OR summary("symptom control") OR summary("symptom assessment") OR summary("symptom monitor*")) |
| S9 | [S1] AND [S7] AND [S8] |

CNKI

| Search | Query |
| --- | --- |
| #1 | 肺肿瘤 + 肺癌 + 非小细胞肺癌 + 小细胞肺癌 |
| #2 | 电子健康病例 + 电子医疗记录 + 远程医学 + 电子 + 数字化 + 远程 + ePRO |
| #3 | 患者报告结局评价 + 患者报告结局 + 患者自我报告 + PRO* + 自我报告 |
| #4 | #2 AND #3 |
| #5 | 症状监测 + 症状管理 + 症状评估 + 症状控制 |
| #6 | #1 AND #4 AND #5 |

WanFang

| Search | Query |
| --- | --- |
| #1 | 主题：（肺肿瘤 OR 肺癌 OR 非小细胞肺癌 OR小细胞肺癌） |
| #2 | 主题：((电子健康病例 OR 电子医疗记录 OR 远程医学 OR 电子 OR 数字化 OR 远程 OR ePRO） AND（患者报告结局评价 OR 自我报告 OR 患者自我报告 OR PRO*）） |
| #3 | 主题：（症状监测 OR 症状管理 OR 症状评估 OR 症状控制） |
| #4 | #1 AND #2 AND #3 |

SinoMed

| Search | Query |
| --- | --- |
| #1 | "肺肿瘤"[常用字段:智能] OR "肺癌"[常用字段:智能] OR "非小细胞肺癌"[常用字段:智能] OR "小细胞肺癌"[常用字段:智能] |
| #2 | "电子健康病例"[常用字段:智能] OR "电子医疗记录"[常用字段:智能] OR "远程医学"[常用字段:智能] OR "电子"[常用字段:智能] OR "数字化"[常用字段:智能] OR "远程"[常用字段:智能] OR "ePRO"[常用字段:智能] |
| #3 | "患者报告结局评价"[常用字段:智能] OR "患者报告结局"[常用字段:智能] OR "患者自我报告"[常用字段:智能] OR "PRO*"[常用字段:智能] OR "自我报告"[常用字段:智能] |
| #4 | ("患者报告结局评价"[常用字段:智能] OR "患者报告结局"[常用字段:智能] OR "患者自我报告"[常用字段:智能] OR "PRO*"[常用字段:智能] OR "自我报告"[常用字段:智能]) AND ("电子健康病例"[常用字段:智能] OR "电子医疗记录"[常用字段:智能] OR "远程医学"[常用字段:智能] OR "电子"[常用字段:智能] OR "数字化"[常用字段:智能] OR "远程"[常用字段:智能] OR "ePRO"[常用字段:智能]) |
| #5 | "症状监测"[常用字段:智能] OR "症状管理"[常用字段:智能] OR "症状评估"[常用字段:智能] OR "症状控制"[常用字段:智能] |
| #6 | ("症状监测"[常用字段:智能] OR "症状管理"[常用字段:智能] OR "症状评估"[常用字段:智能] OR "症状控制"[常用字段:智能]) AND (("患者报告结局评价"[常用字段:智能] OR "患者报告结局"[常用字段:智能] OR "患者自我报告"[常用字段:智能] OR "PRO*"[常用字段:智能] OR "自我报告"[常用字段:智能]) AND ("电子健康病例"[常用字段:智能] OR "电子医疗记录"[常用字段:智能] OR "远程医学"[常用字段:智能] OR "电子"[常用字段:智能] OR "数字化"[常用字段:智能] OR "远程"[常用字段:智能] OR "ePRO"[常用字段:智能])) AND ("肺肿瘤"[常用字段:智能] OR "肺癌"[常用字段:智能] OR "非小细胞肺癌"[常用字段:智能] OR "小细胞肺癌"[常用字段:智能]) |
