# Supplementary material for: The Current Landscape of Remote Digital Symptom Monitoring for Patients With Lung Cancer: Scoping Review
Source: J Med Internet Res. 2026 Mar 24;28:e83666. doi: 10.2196/83666 (PMC13012230; doi:10.2196/83666)
Supplement: Multimedia Appendix 2 [file jmir-v28-e83666-s002.docx]

Appendix 2. Characteristics of Included Studies

| **Author**  **Year**  **Country**  **Study Type**  **Setting** | **Patient/ Treatment Characteristics**  **Treatment status**  **Adherence rate** | **System Components**  **&**  **Symptom monitored** | **System flow**  **Monitor Duration** | **Outcome Measures/Scales** | **Main findings** |
| --- | --- | --- | --- | --- | --- |
| Stover [1]  2025   - USA - Feasibility study - Outpatient | Pop: N=41  Age: Mean=61.5  Gender: Male (n=16, 39%)  Type:   - NSCLC & SCLC - Stage I-IV   Status:   - Receiving outpatient treatment   Adherence rate: Mean=66% | **Moovcare (via smartphone, tablet or PC)**  Components:   - Completion of PROMs; - Alert algorithm; - Follow-up intervention   **Symptom monitored**  NA | - HCPs leading - Patients report weekly via Moovcare; - Study coordinator will remind patients in clinic or via phones after the third day if a survey remained incomplete. - Providers are automatically alerted via email or text message if a concerning symptom is predicted. - Providers followed their usual clinical protocols for managing symptoms - The study coordinator will notify the clinical team if an alert has not been responded to within 48 hours.   Monitor duration:  25 weeks | Primary:   - Feasibility of Moovcare intervention (Participation and enrollment, ePRO completion and alert rates.)   Secondary outcome:   - HRQL (EORTC-QLQ-C30, QLQ-LC13) - Treatment team satisfaction (CAHPS, AHRQ, and PCC–CA-6) | - The median weekly ePRO completion rate was 76% (IQR: 40%-96%) - Alerts occurred in 32/41 (78.0%) of patients, with a mean of 4.2 alerts (95% CI, 2.7-5.69) - HRQOL was largely unchanged, - Satisfaction with provider communication was high. |
| Sewell[2]  2025   - USA - Prospective study - Post discharge | Pop: N=1520  Age: Mean=65  Gender: Male (n=701, 46%)  Type: Not reported  Status:   - Undergoing operations   Adherence rate   - 61% | **Online platform**  Components:   - Completion of PROMs; - A symptom reporting system with alerts; - Notifying HCPs based on alert classification.   **Symptom monitored**  NA | - Nurse leading - Patients complete ePRO daily via platform; - The color-coded grading system provides different responses based on the varying symptom severities reported by patients. - Tailor made suggestions and feedback from nursing team.   Monitor duration:   - 10-14days | Primary:   - Readmissions rates (Univariable analysis) | - Electronic reporting adds the potential to lower the odds of readmission |
| Pongiglione[3]  2025   - Italy - RCT - Outpatient and inpatient oncology | Pop: N=100  Age: Mean=61  Gender: Male (54%)  Type: Not reported  Status:   - Undergoing treatment   Adherence rate   - 83% | **LuCApp (via smartphone)**  Components:   - Completion of PROMs; - Reporting weight and temperature; - Alert system; - Document telephone counseling between HCPs and patients; - Daily suggestions to help better manage the side effect   **Symptom monitored**   - 21 cancer-related side effects - Anxiety, Depression | - Clinicians leading - Patients report every other day; - Clinicians will contact patients when threshold are met. - Every telephone consultation will be documented | Primary:   - HRQL (FACT-L)   Secondary:   - Change of the patients-reported outcome (FACT-L, EuroQoL 5D-5L, HADS) - Usability and satisfaction (CSUQ) - Resource utilization (Descriptive) | - No significant difference in HADS-Depression and EQ-5D-5L scores between groups at both time points. - The use of resources and related costs were lower among LuCApp patients (€2900 vs €3720), not statistically significant (p=0.138) |

Appendix 2. Characteristics of Included Studies (Continued)

| **Author**  **Year**  **Country**  **Study Type**  **Setting** | **Patient/ Treatment Characteristics**  **Treatment status**  **Adherence rate** | **System Components**  **&**  **Symptom monitored** | **System flow**  **Monitor Duration** | **Outcome Measures/Scales** | **Main findings** |
| --- | --- | --- | --- | --- | --- |
| Nuamek[4]  2025   - UK - Feasibility study - Outpatient | Pop: N=19  Age: Mean=66  Gender: Male (41.1%)  Type: SCLC & Stage III NSCLC  Status: undergoing radiotherapy or chemotherapy  Adherence rate: 75.3% (LC group) | **Smartphone and PC**  Component:   - Completion of PROMs; - Automated advice   **Symptom monitored**   - Pain, dyspnea, cough, dysphagia, anorexia, and fatigue - Anxiety | - Clinicians leading - Patients report weekly; - Automated advice based on symptom severity - Patients can raise concerns via 24-hour hotline - Follow-up triggered when threshold is met   Monitor duration:   - 8-12 weeks | Primary:  Feasibility (ePROMs completion rate)  Secondary:  Acceptability (Qualitative interview) | - ePROMs is feasible (completion rate was 69.1%, ranging from 47.4% to 89.5% at each timepoint) - ePROMs is acceptable (all participants reported ease of use) |
| Jing[5]  2025   - China - RCT - Outpatient and inpatient | Pop: N=355  Age:   - n=99 (<60 y) - n=74 ($\geq$60 y)   Gender: Male (n=218, 61.4%)  Type: Stage I, II & III  Adherence rate: 69.1% | **WeChat mini program (via smartphone or PC)**  **Component:**   - Completion of PROMs; - Follow-up via smartphone if threshold is met; - Tailor-made advice; - Mandatory response system   **Symptom monitored**   - Cough, pain, sleep disorders, fatigue, sadness, shortness of breath, and depression. | - Clinicians leading - Patients report at specific intervals; - Automated advice based on symptom severity - Doctors provided standardized management guidance within 24 h when symptom threshold is met.   Monitor duration:   - 12 weeks | Primary:   - Average number of target symptom threshold events at 12 week (MDASI-LC)   Secondary:   - ePRO trajectory changes in symptom score (descriptive) - daily function (MDASI-LC) - QOL (EORTC QLQ-C30) | - ePROMs group shows a significantly lower symptom threshold [0 (0–1) vs. 1 (0–3)], lower symptom scores [adjusted mean difference, −0.527 (95% CI: −0.788 to −0.266)], and higher QOL scores (emotional function: 2.908; 95% CI: 0.600–5.216, P = 0.014; global health: 6.775; 95% CI: 3.967–9.583) |
| Lv [6]  2025   - China - RCT   Home-based | Pop: N=114  Age:   - IG (Mean=61) - CG(Mean=64)   Gender:   - Male (N=68, 59%)   Type:   - I–II NSCLC   Scheduled for Treatment；  83.8% | **Mobil Phone**  Components:   - A symptom reporting system with alerts, - Aerobic and respiratory training exercises, - Educational material.   **Symptom monitored**   - Pain, shortness of breath - Weakness, fatigue, sleepiness, disturbed sleep, being worried, and difficulty remembering - Cough blood, nausea, vomiting, and constipation | - Daily symptom reporting in the resting state for the four core symptoms and triggering alerts to the medical team if the extent was severe (Red alert). - Completing the daily aerobic and respiratory exercises in the app program. - Reading all four aspects of the educational material as a supplement - From discharge to 1-month follow-up (POD 7, 14, 21, 28) | Primary outcome:   - Pulmonary function (Calculation)   Secondary outcome:   - Symptom burden (MDASI-LC) - Aerobic exercise intensity (10-point Borg scale of shortness of breath) - Emergency department visits - App-related safety   Satisfaction with the app (self-made tools) | - Full intervention group with markedly higher PFRR than standard care (79.14% vs. 73.12%; P = 0.017). - APP group with notably reduced interference in general activities, work (1-2 weeks post-discharge), and walking (1 week to follow-up) vs. Standard care (all P < 0.05). - Application group with markedly lower pain, cough, fatigue, and sleep disturbance scores vs. Standard care group at 1 week post-discharge and follow-up (all P < 0.05). - Application group with notably higher Borg scores vs. Standard care on postoperative day 21 (2.84 vs. 1.65, P < 0.001) and day 28 (2.83 vs. 1.15, P < 0.001). |

Appendix 2. Characteristics of Included Studies (Continued)

| **Author**  **Year**  **Country**  **Study Type**  **Setting** | **Patient/ Treatment Characteristics**  **Treatment status**  **Adherence rate** | **System Components**  **&**  **Symptom monitored** | **System flow**  **Monitor Duration** | **Outcome Measures/Scales** | **Main findings** |
| --- | --- | --- | --- | --- | --- |
| Blakely [7]  2025   - USA - Cohort study - Community | Pop: N=910  Age:   - Historical cohort (Mean=67) - Soc cohort (Mean=69)   Gender:   - Historical cohort (Male N=271, 29%) - Soc cohort (Male N=252, 27%)   Type:   - Advanced NSCLC   On Treatment/ Completed Treatment;  36% | **Noona platform (via smartphone, tablet or computer)**  Components:   - Symptom module - Diary function - Secure messaging - Laboratory reports   **Symptom monitored**  NA | - 5-7 days post-pembrolizumab infusion, patients get email/app alert to complete symptom questionnaire. - Tailored to treatment and symptoms, with rule-based alerts for moderate/severe issues. - If not completed in 3 days, 1 reminder sent; no response triggers call on days 8-10; expires day 7. - Patients can complete symptom questionnaires anytime beyond scheduled prompts. - Team (nurse/doctor/pharmacist) reviews submissions using triage algorithms (NCCN/ONS/ASCO guidelines). - Treatment initiation to 12 months post-profile | Primary:   - rwToT (Kaplan-Meier, Cox model);   Secondary:   - rwTTNTD, HCRU (Poisson regression); Noona usage patterns | - No significant difference in rwToT (historical: 4.4 mo, SoC: 4.1 mo; aHR 0.9, p=0.14) or rwTTNTD; - HCRU similar; 43% established Noona profile, 63% used it, mostly for lab results (52%) |
| Yang [8]  2025   - Singapore - RCT - Cancer centers | Pop: N=239  Gender:   - CG Male: N=73, 61.3% - IG Male: N=69, 57.5%   Type   - Stage 4 solid tumor   On going treatment  66% | **Phone-based**   - Completion of PROM - Further assessment via phone call - Tailored follow-up   Referral when necessary  **Symptom monitored**   - NA | - Nurses leading - Weekly IPOS complete via phone invitation - Nurse follow-up when necessary - Referral when necessary   Monitor duration   - 16 weeks | Quality of life (FACT-G) | - No significant difference in overall FACT-G score; - Better physical well-being in SPARKLE group (baseline-adjusted difference 1.9, p=0.01); - No difference in palliative care referrals or healthcare utilization |
| Yu [9]  2024   - China - Cohort study   Oncology department | Cohort 1: N=39   - Age (Mean=59.50) - Male (N=24, 61%)   Cohort 2: N=223   - Age: (Mean=53.90) - Male (N=93, 41%)   Cohort 3: N=775   - Age (Mean=52.74) - Male (N=273, 35%)   Type:   - Postoperative lung cancer patients.   On Treatment/ Completed Treatment;  63.2%-84.8% | **Mobile-phone based**   - Data collection using ePROM - Assessment Scheduling - Reminder Mechanism - Alert System   Clinician Response  **Symptom monitored**   - Pain, cough, shortness of breath, disturbed sleep, fatigue, drowsiness, walking difficulty, and activity limitation - Distress | -Data collected via WeChat Mini program at home/hospital.  -Nonreporting triggered 7 PM reminders and calls after 3 missed assessments.  -Alerts sent to clinicians for pain/distress≥7, followed by guideline-based patient contact.   - Pre-op baseline, daily during hospitalization and first month post-discharge, weekly for months 2-3, monthly for months 4-12. | Primary outcome   - ePROM validation (ePSA-Lung)   Symptom burden (MDASI-LC). | - The ePSA-Lung showed excellent internal consistency (Cronbach’s ɑ = 0.91) and test-retest reliability (ICCs, 0.86–0.95). - Pre-surgery compliance: 99%; in-hospital: 62.6%-88.8%; post-discharge: daily 68.5%-84.8%, weekly 72.9%-81.9%, monthly 63.2%-76.4%. |

Appendix 2. Characteristics of Included Studies (Continued)

| **Author**  **Year**  **Country**  **Study Type**  **Setting** | **Patient/ Treatment Characteristics**  **Treatment status**  **Adherence rate** | **System Components**  **&**  **Symptom monitored** | **System flow**  **Monitor duration** | **Outcome Measures/Scales** | **Main findings** |
| --- | --- | --- | --- | --- | --- |
| Mooney [10]  2024   - USA - RCT - Oncology department | Pop: N=757  Age:   - Group 1 (Mean=59.4) - Group 2 (Mean=58.5) - Group 3 (Mean=58.7) - Group 4 (Mean=59.8) - Group 5 (Mean=59.4)   Gender:   - Group 1 (Male N=54, 7%) - Group 2 (Male N=56, 7%) - Group 3 (Male N=59, 8%) - Group 4 (Male N=59, 8%) - Group 5 (Male N=65, 8%)   Type   - Every types of cancer with a life expectancy of 3 months or greater; - Beginning a chemotherapy course planned for at least 3 cycles. - No concurrent radiation therapy;   Scheduled for Treatment;  85.6% | **Phone-based**   - Daily Symptom Monitoring - Automated Self-Management Coaching - Nurse Practitioner Follow-Up   Decision Support System  **Symptom monitored**  NA | - Nurse leading - Patients report symptoms daily via SCH interactive voice-response tool. - Automated self-management coaching delivered during calls (groups 1, 2, 5). - Nurse practitioners (NPs) receive alerts for moderate-to-severe symptoms and follow up (groups 3, 4, 5), with decision support in groups 4 and 5. - Data analyzed for symptom burden reduction.   Daily during chemotherapy, until treatment completion or 6 months. | Primary outcome  Symptom burden (Symptom Care at Home system) | - A multi-component digital approach to cancer symptom management may offer optimal symptom burden reduction. |
| Dai [11]  2024   - China - RCT   Oncology department | Pop: N=112  Age:   - IG (Mean=51.6) - CG (Mean=51.2)   Gender:   - IG (Male N=22， 19%) - CG (Male N=19， 16%)   Type   - Stage I-IIIA lung cancer patients   On Treatment/ Scheduled for Treatment;  IG: 96.4%; CG: 96.5% | **Personal electronic devices**   - ePRO system: Real-time symptom reporting and alerts for scores ≥4.   Surgeon response: Guided by clinical guidelines within 24 hours.  **Symptom monitored**   - Pain, fatigue, disturbed sleep, shortness of breath, and coughing | - Symptoms reported via ePRO, with alerts triggering surgeon response in ePRO group. - Responses during hospitalization occurred during daily ward rounds - Post discharge responses used text messages, voice messages, or calls. - Surgeons had a 24-hour response window   Assessment schedule   - Daily during hospitalization, twice weekly until 4 weeks post discharge, then at 3, 6, 9, and 12 months post discharge. | Primary outcome  -Number of symptom threshold events at 12 months post discharge (MDASI-LC)  Secondary outcome  -Composite physical and affective interference scores (MDASI-LC) | -Primary: 18 patients (32.7%) generated 50 symptom threshold events, resulting in 34 alerts.  -Secondary: At 12 months post-discharge, IG reported significantly fewer symptom threshold events compared to the usual care group (median [IQR], 0 [0-0] vs. 0 [0-1]; p = 0.040).   - -From 1 to 12 months post-discharge, IG reported lower composite scores for physical interference (estimate: –0.86; 95% CI: –1.32 to –0.39) and affective interference (estimate: –0.70; 95% CI: –1.14 to –0.26) over time compared to the usual care group. |

Appendix 2. Characteristics of Included Studies (Continued)

| **Author**  **Year**  **Country**  **Study Type Setting** | **Patient/ Treatment Characteristics**  **Treatment status**  **Adherence rate** | **System Components**  **&**  **Symptom monitored** | **System flow**  **Monitor duration** | **Outcome Measures/Scales** | **Main findings** |
| --- | --- | --- | --- | --- | --- |
| Friis [12]  2024   - Denmark - RCT - Oncology departments | Pop: N=494  Age:   - IG (Mean=68) - CG (Mean=70)   Gender:   - IG (Male N=98， 19%) - CG (Male N=124， 25%)   Type:   - NSC or SC LC patients - Stage III/IV disease treated with palliative intention   On Treatment/ Completed Treatment;  76% | **Web-based**   - Electronic questionnaire - Nurse alert system   **Symptom monitored**   - Dyspnea, pain, fatigue, appetite loss, coughing, and hemoptysis - Fever, hoarseness, facial swelling, sense of a growing tumor, and weight loss | - Nurse leading - Patients completed weekly questionnaires; - Nurses reviewed reports; - Contacted patients by phone if thresholds exceeded; - Adjusted supportive care   Monitor duration   - Weekly monitor, median duration of patient participation was 24 weeks (minimum 1, maximum 104) | - Overall survival (Kaplan-Meier method); - HRQoL (EORTC QLQ-C30, QLQ-LC13, HADS, EQ-5D-5L); - Progression-free survival; - ECOG PS at progression; - Second-line treatment rates | - No significant OS improvement (HR, 0.93; P=.53); - Modest HRQoL improvements (e.g., EORTC QLQ-C30 Physical Functioning: 3.1 points, P=.04, below MID); - Subgroup benefits with carboplatin/vinorelbine (HR, 0.67; P=.04) |
| Alix [13]  2024   - USA - Qualitative study - Cancer department | Pop: N=16  Age: Mean=56.1  Gender:   - Male (N=4, 25%)   Type   - All stages and types of cancer   Completed Treatment；  45% | **Web-based**   - ePRO platform: symptom surveys, - Email reminders, - Clinician alerts (active arm)   **Symptom monitored**  **NA** | - Surveys sent via email over 90 days post-discharge; - Reminders after missed surveys; - The active arm had clinician alerts   Monitor duration   - Not specified | Primary outcome   - Patient-perceived barriers and facilitators to ePRO use | - Barriers include: 1. Postoperative patient physical and mental health, 2. Lack of access to email and poor internet connectivity, 3. Lack of clarity on ePROM use in routine clinical care, and 4. Symptom item redundancy. - Facilitators include: 1. Ease of the ePROM assessment completion, 2. Engagement with the surgical care team on ePRO use, and 3. Increased awareness of symptom experience through ePRO use. |
| Arriola [14]  2024   - Spain - Prospective study - Clinics practice | Pop:   - Patients N=110 - HCP N=30   Age Not Reported  Gender   - Male (N=61, 55%)   Type  -Advanced lung cancer or HER-2 positive breast cancer  On Treatment/ Scheduled for Treatment;  77%~81% | **Personal electronic devices**   - Symptom self-reporting - HCP alerts for critical symptoms - Direct messaging - Self-management materials - Tailored content by F. Hoffmann-La Roche Ltd on the Kaiku Health platform   **Symptom monitored**  NA | - Nurse leading - Patients report symptoms via the DPM tool. - Hcps receive daily digest emails and immediate alerts for severe symptoms (grade 3 or ≥2-grade change) within 15 minutes, prioritizing review and management.   Monitor duration   - Weekly prompted symptom questionnaires, with ad hoc reporting available; - Up to 15 months | Primary outcome   - Patient adoption (threshold: ≥60%) and adherence to weekly symptom reporting at week 6 (threshold: ≥70%)   Secondary outcome   - User experience (NPS, CSAT, and CES) - Clinical impact (HCP care impact questionnaire) - Patient communication (HCP care impact questionnaire) - HRQoL (Wilcoxon signed rank test on questionnaire data) | - Primary: Both adoption and adherence were above the threshold for positive endpoints (60% and 70%, respectively) - Secondary: Customer satisfaction and effort scores for patients were 76% and 82%, respectively, and 83% and 79% for hcps; Most patients and hcps felt that the DPM tool covered/mostly covered symptoms experienced (71% and 75%), was educational (65% and 92%), and improved patient-HCP conversations (70% and 83%) and cancer care (51% and 71%) |

Appendix 2. Characteristics of Included Studies (Continued)

| **Author**  **Year**  **Country**  **Study Type Setting** | **Patient/ Treatment Characteristics**  **Treatment status**  **Adherence rate** | **System Components**  **&**  **Symptom monitored** | **System flow**   - **Monitor duration** | **Outcome Measures/Scales** | - **Main findings** |
| --- | --- | --- | --- | --- | --- |
| Payne [15]  2023   - UK - Prospective study   Clinics practice | Pop: N=100 (LC=78)  Age: Mean=68.5  Gender:   - Male (N=36, 36%)   Type  -All stages and types of lung cancer or breast cancer  On Treatment/ Completed Treatment;  93% | **Personal electronic devices**   - Symptom reporting - Color-coded advice alerts   Access via personalized links  **Symptom monitored**  NA | - Nurse leading program - Patients receive a text/email link before appointments - complete ePROMs - get color-coded advice - clinicians review responses on a separate platform (DrDoctor®) before consultations   Monitor duration   - Before each clinic visit (day before first appointment or three days prior to follow-ups) | Primary outcome   - Patient experience and clinician experience (PREM, Clinician experience questionnaire with Likert scale and free-text comments.)   Secondary outcome   - Usability (PREM) - Communication (PREM) - Patient involvement (PREM) - Clinical decision-making (PREM)   Consultation focus (PREM) | - Patients reported that MyChristie-MyHealth was easy to understand, with nearly all patients indicating that they could complete it in a timely manner and found it easy to follow. - Improved communication with the oncology care team (82%). - Assisted the care team in participating in their own care (88%). - More than half of the doctors believe that ePROMs help improve communication with patients and reflect a more patient-centered approach. |
| Patt [16]  2023   - USA - Cohort study   Community | Pop: N=559  Age:   - IG Mean=74.47 - CG Mean=74.06   Gender   - IG (Male N=119, 21%) - CG (Male N=118, 21%)   Type:   - Patients receiving treatment for metastatic cancer.   On Treatment;  55% | **Navigating Cancer’s ePRO software system**   - Modified PRO-CTCAE item library - Real-time alert module   Nurses follow-up  **Symptom monitored**  **NA** | - Nurse leading - Patients weekly report via email or text - Real-time alerts triggered when moderate to severe symptom identified - Nurses follow-up   Monitor duration   - 5 months | Primary outcome   - Total adverse events (aes: hospitalizations, ER visits, deaths) and total cost of care.   Secondary outcome   - Hospitalization rate - ER visit rate - Death rate   Cost breakdown by service type. | - Mean total AEs were lower in the study group compared with control (0.98 v 1.41; P 5 .007). - The total cost of care was reduced by an average of $1,146 per member per month. |
| Parikh [17]  2023   - USA - RCT   Clinical setting | Pop: N=108  Age:   - IG Mean=59 - CG Mean=61   Gender:   - IG a (Male N=22, 20%) - IG b (Male N=21, 19%) - CG (Male N=16, 14%)   Type   - Stage IV GI or lung cancers   On Treatment;  77.8% | **Phone-based**   - Weekly text message-based symptom surveys. - Passive activity monitoring with a Fitbit accelerometer. - Clinician dashboards summarizing patient-generated health data.   Optional active choice text prompts for patients to discuss worsening symptoms/function.  **Symptom monitored**  NA | Patients received weekly surveys and wore accelerometers   - Data were collected remotely, summarized in dashboards, and provided to clinicians before appointments - Active choice prompts were sent to some patients before visits   Monitor duration   - Weekly for symptom surveys; daily for accelerometer data (synced periodically). - Up to 6 months. | Primary outcome (1-5 Likert scale)   - Patient-perceived clinician understanding of symptoms and functional status at 6 months   Secondary outcome   - Responses to the same questions at 3 months - Adherence to PGHD collection - Responses about communication and burden   Hospitalization/emergency room utilization and palliative care visits | - - No difference in clinician understanding (symptoms: 4.5 vs. 4.5, P = 0.87; functional status: 4.5 vs. 4.3, P = 0.31) or functional status (4.5 vs. 4.3; P = 0.31)   - Patient adherence (weekly symptom reports: 64%; daily activity monitoring: 53%). - Intervention patients in the PROStep versus PROStep 1 active choice arms reported low burden from wearing the accelerometer (2.7 [1.3] vs. 2.1 [1.3], P = 0.15) and completing surveys (2.1 [1.2] vs. 1.9 [1.3], P = 0.44). |

Appendix 2. Characteristics of Included Studies (Continued)

| **Author**  **Year**  **Country**  **Study Type Setting** | **Patient/ Treatment Characteristics**  **Treatment status**  **Adherence rate** | **System Components**  **&**  **Symptom monitored** | **System flow**  **Monitor duration** | **Outcome Measures/Scales** | **Main findings** |
| --- | --- | --- | --- | --- | --- |
| Ma [18]  2023   - USA - Prospective study - Department of Radiation Oncology | Pop: N=19  Age: Mean=59  Gender: Not reported  Type   - Patients with gastrointestinal, lung, or head and neck cancers undergoing chemoradiation therapy.   On Treatment;  100% | **Website or mobile app**   - Web-based symptom self-reporting via myUCLAHealth. - Automated email alerts   Symptom reports provided to clinicians at visits  **Symptom monitored**  **NA** | - Nurse leading - Patients self-report symptoms via WBI; - Alerts sent to nurses for severe/worsening symptoms - Nurses record actions - Reports are given to clinicians at visits   Monitor duration   - Biweekly (every 3-4 days) during CRT; weekly for 3 months post-CRT; monthly for 3 additional months.   Up to 6 months. | Primary Outcome   - Feasibility of symptom self-reporting via WBI (adherence rate)   Secondary outcome   - Clinician response to alerts (Tracked via standardized nursing forms.) - patient satisfaction (Exit survey, 1-7 scale) - acute hospital encounters (Medical records.)   HR-QOL (PROMIS surveys). | - Feasible with high patient satisfaction (100% reported care improved); - low survey completion (26% during CRT, 33% post-CRT); - 87% on-treatment alerts addressed within 4 days, - 60% post-CRT alerts within 7 days;   - 26% had acute hospital encounters |
| Lee [19]  2023   - Korea - RCT   Medical center | Pop: N=213  Age:   - IG Mean=56 - CG Mean=55.8   Gender:   - IG (Male N=50, 23%) - CG (Male N=23, 10%)   Type   - Patients with breast, lung, head and neck, esophageal, or gynecologic cancers.   Scheduled for Treatment；  43% | **Mobile app**   - ePRO-CTCAE mobile app for symptom monitoring - Graphical summaries for patients - Web dashboard and printed reports for physicians   Educational material and app training  **Symptom monitored**  **NA** | - Patients report symptoms weekly via the app - Data are sent to a web dashboard - Physicians access summaries during practice - No alerts are triggered   Monitor duration   - Weekly (every 7 days) for 8 weeks. | Primary Outcome   - Patient participation in symptom management (Custom 10-item questionnaire)   Secondary Outcome   - HRQoL (EORTC-QLQ-C30)   Unplanned clinical visits (Self-reported via survey) | - The intervention group reported better outcome in patient participation in symptom management than the control group at 8 weeks (mean scores of 8.5 vs. 8.0; P = 0.01). - There were no significant differences between the groups in Quality of life (P = 0.88) and unplanned clinical visits (P = 0.39–0.76). |
| Schougaard[20]  2023   - Denmark - Qualitative study   Oncology department | Pop=18  Type   - Nurses using ProWide intervention.   On Treatment；  NA | **Web-based intervention**   - PRO data collection - PRO-based algorithm - PRO-based graphic overview - Reminders are sent to those not responding. - Alert system to the clinical department.   Nurses follow up  **Symptom monitored**  **NA** | - Nurse leading - Patients complete ePRO and CT scan weekly - Nurses reviewed PRO reports daily - Respond to the red alerts - Contacted patients via phone if necessary   Monitor duration   - NA | Nurses’ experiences and barriers | - ProWide supported decision-making by identifying issues and monitoring changes; - Barriers included busy schedules, physical distance, and patient unresponsiveness |

Appendix 2. Characteristics of Included Studies (Continued)

| **Author**  **Year**  **Country**  **Study Type Setting** | **Patient/ Treatment Characteristics**  **Treatment status**  **Adherence rate** | **System Components**  **&**  **Symptom monitored** | **System flow**  **Monitoring duration** | **Outcome Measures/Scales** | **Main findings** |
| --- | --- | --- | --- | --- | --- |
| Wujcik [21]  2022   - USA - Prospective study - Cancer center | Pop: N=282  Age: Mean=58  Gender:   - Male (N=41, 14%)   Type:   - Diagnosed with breast cancer, lung cancer, ovarian cancer, multiple myeloma, or acute myeloid leukemia.   Scheduled for Treatment;  88% | **Personal electronic devices**   - Carevive cloud-based platform - Weekly symptom surveys - Real-time provider alerts - Self-management guidelines - Phone calls for severe symptoms   **Symptom monitored**  **NA** | - Patients complete weekly surveys via the platform - Data integrate into the EMR - Providers receive real-time alerts for severe symptoms - No alerts for mild/moderate symptoms   Monitor duration   - Weekly (every 7 days) for as long as treatment continues | Primary Outcome   - Feasibility (Enrollment numbers, survey completion rates)   Secondary outcome   - Symptom alerts (PRO-CTCAE symptom survey) | - 2,860/3,248 surveys completed (88%). - 152/282 patients (54%) had symptom reports triggering provider alerts. - Commonly Reported Symptoms: Fatigue (84%), pain (54%), decreased appetite (52%), insomnia (51%), nausea (50%). - Alert-Triggering Symptoms: Pain (42%), fatigue (32%), insomnia (27%). |
| Dai [22]  2022   - China - RCT   Hospital | Pop: N=134  Age:   - IG (Mean=51.6) - CG (Mean=51.7)   Genders:   - IG (Male N=26, 19%) - CG (Male N=24, 17%)   Type:   - Stage I-IIIA LC   Scheduled for Treatment;  66% | **Mobile app**   - Electronic symptom monitoring - Real-time alerts   Surgeon response within 24 hours  **Symptom monitored**   - Pain, fatigue, disturbed sleep, shortness of breath, and coughing | - Patients report symptoms via ePRO - intervention group triggers alerts for scores ≥ 4 to surgeons for response - control group data collected but no alerts or surgeon access   Monitor duration   - Daily during hospitalization, twice weekly for 4 weeks post discharge. | Primary outcome   - Number of symptom threshold events at discharge (MDASI-LC)   Secondary outcome   - Symptom threshold events at 4 weeks post discharge (MDASI-LC) - Composite symptom score (MDASI-LC) - Physical and affective interference scores (MDASI-LC) - QoL score (SIQOL) - Revisit rate   Complications. | - The intervention group reported fewer symptom threshold events than the control group (median [interquartile range], 0 [0-2] v 2 [0-3]; P 5 .007). - The intervention group had a lower complication rate than the control group (21.5% v 40.6%; P 5 .019). |
| Girgis [23]  2022   - Australia - Mixed-methods study   3 hospitals in SWSLHD, Sydney | Pop=48  Age: (Mean=69)  Gender:   - Male (N=29, 60%)   Type:   - All types of LC   On treatment;  61% | **iPad (first time), Web-based**   - Physical evaluation (ESAS). - Psychosocial evaluation (DT). - Automated email alerts to notify care providers of unresolved issues.   Tailored patient self-management resources.  **Symptom monitored**  **NA** | - Care coordinator leading - Monthly assessments (in-clinic or remote); - Care coordinators reviewed eMR results, actioned alerts, - Offered intervention or referrals if necessary.   Monitor duration   - Five months | - Reach, effectiveness (ED/Cancer assessment units, CAU visits, referrals; multivariable logistic regression), - Adoption (ORIC, HCP surveys), - Implementation (alerts actioned),   Maintenance (staff engagement; field notes, interviews) | - High reach (61% onboarded); - Fewer CAU visits (p=0.035), - More referrals (p<0.0001); - Staff confidence improved (p<0.005); - 95.6% alerts actioned; - Sustained coordinator engagement |

Appendix 2. Characteristics of Included Studies (Continued)

| **Author**  **Year**  **Country**  **Study Type Setting** | **Patient/ Treatment Characteristics**  **Treatment status**  **Adherence rate** | **System Components**  **&**  **Symptom monitored** | **System flow**  **Monitoring duration** | **Outcome Measures/Scales** | **Main findings** |
| --- | --- | --- | --- | --- | --- |
| Zhang [24]  2022   - China - RCT - 28 tertiary care hospitals in China | Pop: N=278  Age  IG (Mean=57.6)  CG (Mean=60.1)  Gender  IG (Male N=106, 38%)  CG (Male N=100, 35%)  Type   - Patients receiving cancer immunotherapy   On treatment;  92.7% | **Mobile application**   - Weekly questionnaire of common symptoms, - Image recognition for irAE grading, - Automated advice, - Alerts for severe irAE   **Symptom monitored**  NA | - Patients completed weekly questionnaires, - Uploaded examination images; - Automated advice for grade 1-2 irAEs; - Alerts and team follow-up for grade 3-4 irAEs   Monitor duration   - Weekly report - Up to 6 months | - Incidence of severe irAEs (NCI-CTCAE v5.0); - ED visits; - QOL (EORTC QLQ-C30); - Treatment discontinuation; - Death rates; - Follow-up time | - ePRO reduced severe irAEs (20.6% vs 33.6%; HR, 0.51; P=.01), - ED visits (16.3% vs 29.9%; HR, 0.46; P=.01), - Treatment discontinuation (3.6% vs 11.0%; HR, 0.30; P=.02), - Improved QOL (74.2 vs 64.7; P=.001), - Reduced follow-up time (8.2 vs 36.1 min; P<.001) |
| Mody [25]  2021   - USA - Cohort study - Community | Pop: N=593  LC population:  Age (Mean=64.4)   - Male N=49, 8%   Type   - All stages and types of cancer.   On treatment;  88.6% | **Web-based/ Interactive Voice Recording**   - Weekly electronic PRO surveys - Automated alerts to clinicians for concerning symptoms - Clinician response   **Symptom monitored**  NA | - Patients complete weekly pro surveys - Alerts triggered for concerning symptoms are sent to clinicians - Clinicians respond with interventions - Control arm uses educational materials without alerts   Monitor duration   - Weekly for up to 12 months | Primary Outcome   - Survey completion rates (PRO-CTCAE) - symptom occurrence   Secondary Outcome   - QOL (EORTC QLQ-C30) - patient satisfaction (Custom 5-point scale survey) - clinician management strategies (Recorded interventions post-alert) | - Of delivered weekly PRO surveys over 12 months, 91% were completed. - Nearly all (97%) patients reported concerning symptoms during participation. - Pain was the most frequent and longest lasting symptom. - 87% of clinicians recommend using ePRO monitoring. |
| Cheng[26] 2021   - China - Prospective study   Tertiary lung cancer center | Pop: N=589  Age: (Mean=59.0)  Gender:   - Male: N=216, 36%   Type:   - Any type and stage of LC patients   Scheduled for treatment;  71.3% | **Web-based**   - Completion of PROM   Reminder of PROM completion  **Symptom monitored**   - Pain, and cough | - Surveys at 2, 4, 6, 8, 12 weeks post-discharge - Telephone follow-up when no response   Monitor duration   - 2, 4, 6, 8, 12 weeks post-discharge | - Pain severity (Numeric rating score)   Cough (Cough visual analog scale) | - Pain and cough severity decreased over 12 weeks; - Factors like female gender, age >60, thoracotomy, and prolonged surgery/drainage affected recovery. |
| Zylla [27]  2020   - USA - Prospective study   Community | Pop: N=80  Age (Mean=62)   - Male (N=27, 33%)   Type   - Stage IV non-hematologic cancers   On Treatment;  83% | **Web-based**   - Epic Mychart surveys - Automated symptom alerts - Triage follow-up   Alternative completion methods  **Symptom monitored**  **NA** | - Patients receive surveys via MyChart every 14 days - Incomplete surveys followed up by phone/paper - Severe symptoms trigger RC review and triage if needed - Alerts triggered if threshold reached   Monitor duration   - Every 14 days over 12 weeks | Primary Outcome   - Feasibility of ePRO integration into Epic MyChart (survey response rates and system implementation success）   Secondary Outcome   - Symptom burden (PRSM) - Patient satisfaction (satisfaction questionnaires) - Clinician satisfaction (satisfaction questionnaires) | - 183 surveys (66%) completed; 68% via MyChart, 25% on paper, 7% by phone call from research coordinator. - Patients and clinicians found the ePRO system efficient and helpful for managing distressing symptoms; recommended for routine oncology care. |

Appendix 2. Characteristics of Included Studies (Continued)

| **Author**  **Year**  **Country**  **Study Type Setting** | **Patient/ Treatment Characteristics**  **Treatment status**  **Adherence rate** | **System Components**  **&**  **Symptom monitored** | **System flow**  **Monitoring duration** | **Outcome Measures/Scales** | **Main findings** |
| --- | --- | --- | --- | --- | --- |
| Iivanainen [28]  2020   - Finland - Prospective study - Outpatient | Pop: N=37  Age (Mean=61.7)  Gender:   - Male (N=27, 72%)   Type   - Advanced cancer patients receiving anti-PD-(L)1 therapy in outpatient settings.   On Treatment/ Scheduled for Treatment;  86% | **Smartphones and computers**   - Kaiku Health ePRO tool - Severity grading algorithm - Urgency alert system - Email notifications/reminders   **Symptom monitored**  **NA** | - Patients receive weekly email notifications to complete symptom questionnaires - Unanswered surveys trigger daily reminders for 6 days - Severe symptoms (grade 3+) or worsening (grade 0 to 2) generate alerts - Care team responds within 3 days   Monitor duration   - Weekly up to 6 months or until disease progression (average 0.58-1.27 questionnaires per week). | Primary Outcome   - Feasibility of ePRO follow-up (patient-reported symptoms, severity, and compliance)   Secondary Outcome   - Spectrum of symptoms - Alert etiology - Symptom correlations with treatment benefit | - Grade 1 symptom Fatigue (28%), Itching (13%). - Grade 2 symptom Loss of Appetite (12%), Nausea (12%). - Grade 3-4 symptom Cough (6%), Loss of Appetite (4%). - Common Alert Triggers: Loss of appetite and shortness of breath. |
| Friis [29]  2020   - Denmark - Feasibility study - Oncology department | Pop: N=20  Age: (Mean=70.5)  Gender:   - Male (N=13, 65%)   Type   - Stage IV lung cancer.   On Treatment;  69% | **Web-based**   - Symptom-specific threshold algorithm - Notification list - Patient comments function   **Symptom monitored**   - AmbuFlex ePRO (EORTC Item Library–based): dyspnea, pain, fatigue, appetite loss, cough, hemoptysis, fever, hoarse voice, facial swelling, tumor growth sensation, weight change, overall health | - Patients report symptoms weekly via the internet - Responses exceeding thresholds trigger notifications - Nurses review daily and contact patients if needed   Monitor duration   - Weekly for 4 weeks during the feasibility test. | Primary Outcome  Feasibility, usability, and acceptability of the ePRO | - The tool demonstrates good feasibility, acceptability, and usability. |
| Schmalz [30]  2020   - Germany - Mix method study - 10 clinics (community to university hospitals) | Nurse N=13  Physicians N=11  Patients N=27  Type:   - Advanced/metastatic NSCLC; second-line CIT (20% atezolizumab);   On treatment/ Completed treatment;  80% (Patients) | **Web-based**   - Kaiku Health CIT+ module: 18-item symptom questionnaire (PRO-CTCAE), - Direct messaging - Educational material - Symptom alerts   **Symptom monitored**  **NA** | - Weekly symptom reporting - HCP alerts for severe symptoms - Direct messaging; onboarding(≤30 min/patient)   Monitor duration   - Weekly report - Up to 3 months | - User satisfaction, communication, quality of care, efficiency (Likert-scale surveys, HCP interviews); - Tool usage (system statistics) | - High user satisfaction; - Improved communication, care quality, efficiency; reduced visits/calls; workflow integration challenges noted |
| Gustafson [31]  2017   - USA - Pooled analysis of two randomized clinical trials   Multicenter oncology setting | Pop: N=217  Age:   - Cohort 1 Mean=62.53 - Cohort 2 Mean=62.73   Gender:   - Cohort 1 (Male N=47, 21%) - Cohort 2 (Male N=49, 22%)   Type   - Stage IIIA, IIIB, or IV   On Treatment;  35% | **Web-based**   - CHESS system: caregiver-focused website with symptom reporting (modified ESAS, 10 items);   Clinician Report (CR) for alerts (threshold ≥7 on 0-10 scale)  **Symptom monitored**   - Fatigue, pain, nausea, appetite loss, constipation, diarrhea, dyspnea - Depression, anxiety, distress | - Nurse leading - Caregivers are prompted to complete report patients’ symptom when logging into CHESS - Caregivers completed check-ins every 7 days via CHESS; - CHESS+CR group: alerts sent to clinicians for threshold symptoms (≥7) via email/fax/phone   Monitor duration   - Up to 12 months | - Proportion of improved threshold symptoms (modified ESAS); - Proportion of threshold symptoms reported (modified ESAS) | - CHESS+CR group had higher proportion of improved threshold symptoms (53.0% vs 26.16%, P<.001); - CHESS+CR caregivers reported fewer threshold symptoms (7.7% vs 14.4%, P<.001), possibly due to response bias |

Appendix 2. Characteristics of Included Studies (Continued)

| **Author**  **Year**  **Country**  **Study Type Setting** | **Patient/ Treatment Characteristics**  **Treatment status**  **Adherence rate** | **System Components**  **&**  **Symptom monitored** | **System flow**  **Monitoring duration** | **Outcome Measures/Scales** | **Main findings** |
| --- | --- | --- | --- | --- | --- |
| Mooney [32]  2017   - USA - RCT - Outpatient | Pop: N=358  Age:   - IG (Mean=54.77) - CG (Mean=56.79)   Gender:   - IG (Male N=45, 12%) - CG (Male N=43, 12%)   Type:   - Scheduled for at least three months of chemotherapy.   On treatment;  75.2% | **Telephone-based**   - Daily symptom monitoring - Automated self-management coaching - Automated alerts - Nurse practitioner (NP) follow-up with guideline-based decision support system (DSS)   **Symptom monitored**  **NA** | - Patients report symptoms daily via phone - SCH group receives tailored coaching and alerts for severe symptoms - NPs follow up within 4 hours using DSS and HER - UC group reports without feedback   Monitor duration   - Daily throughout chemotherapy course or up to 6 months | Primary Outcome   - Symptom severity across all symptoms and number of severe, moderate, mild, and no symptom days (measured by daily symptom reports)   Secondary Outcome   - Individual symptom severity (mixed effects linear modeling) | - SCH participants had significantly less symptom severity across all symptoms (P < 0.001). - The relative symptom burden reduction for SCH participants was 3.59 severity points (P < 0.001), roughly 43% of CG. - All individual symptoms, except diarrhea, were significantly lower for SCH participants (P < 0.05). |
| Denis [33]  2017   - France - RCT - Multicenter | Pop: N=121  Age:   - IG (Mean=65.2) - CG (Mean=64.3)   Gender:   - IG (Male N=41, 33%) - CG (Male N=40, 33%)   Type   - Nonprogressive SCLC or NSCLC   Completed Initial Treatment;  90% | **Web-based**   - Weekly self-reported weight and 11 symptoms - Electronic form for reporting - Dynamic alert system - Graphical format for data presentation   **Symptom monitored**   - Appetite loss, fatigue, pain, cough, depression, dyspnea, fever, facial swelling, lump, voice change, hemoptysis, weight change | - Clinician leading - Patients report weekly via electronic form - Data sent immediately to medical team - Alerts trigger nurse phone call for verification - Oncologist contacts patient if confirmed - Clinical visit/imaging within 8 days if relapse/dangerous condition suggested   Monitor duration   - Weekly report up to 6 months | Primary Outcome   - Overall survival (from random assignment to death or last assessment)   Secondary Outcome   - Performance status at first relapse (FACT-L) - QoL (FACT-L) | - Median OS: 19.0 months (experimental) vs 12.0 months (control), P=.001, hazard ratio=0.32 - 6-month QoL: 80.6% stable/improved (experimental) vs 58.6% (control), P=.04 - 72.4% of relapses detected between visits (experimental) vs 32.5% (control), P<.001 - Reduced CT scans in experimental arm |
| Basch [34]  2016   - USA - RCT   Hospital | Pop: N=766  Age:   - IG (Mean=61) - CG (Mean=62)   Gender:   - IG (Male N=184, 24%) - CG (Male N=138, 18%)   Type   - Cancer patients receiving chemotherapy.   Scheduled for treatment;  76% | **Web-based**   - STAR (Symptom Tracking and Reporting) web-based system - 12 symptom questions - 5-point scale - Automated email alerts   Printed reports  **Symptom monitored**  **NA** | - Ongoing symptom reporting (clinic visits or remotely) - Alerts for severe/worsening symptoms - Clinician review - Usual care comparison   Monitor duration   - Weekly (computer-experienced, encouraged remotely); at clinic visits (computer-inexperienced). | Primary Outcome   - Change in HRQL at 6 months (EuroQol EQ-5D Index)   Secondary Outcome   - Survival at 1 year - quality-adjusted survival - ER visits   hospitalizations. | - HRQL Improvement - Intervention Group: 34%, Usual Care: 18%; HRQL Worsening - Intervention Group: 38%, Usual Care: 53% (P < .001). - Fewer ER visits. |

Appendix 2. Characteristics of Included Studies (Continued)

| **Author**  **Year**  **Country**  **Study Type Setting** | **Patient/ Treatment Characteristics**  **Treatment status**  **Adherence rate** | **System Components**  **&**  **Symptom monitored** | **System flow**  **Monitoring duration** | **Outcome Measures/Scales** | **Main findings** |
| --- | --- | --- | --- | --- | --- |
| Strasser [35]  2016   - Switzerland - Cluster-randomized controlled trial - Outpatient oncology setting | Pop: N=264   - CG n=119 - IG n=145   Age:   - CG Mean=67.3 - IG Mean=65.1   Gender:  Male   - CG N=72, 27% - IG N=94, 35%   Type:   - All types of cancer - Receiving palliative chemotherapy   Palliative care;  38% | **Hand-held computer**   - E-MOSAIC on PALM device. - Longitudinal monitoring sheet (LoMoS) contains patients’ symptoms, clinical data, and medications.   **Symptom monitored**  **NA** | - Nurse leading. - Patients completed E-MOSAIC weekly before visits; - LoMoS sheet printed and given to oncologists in intervention arm. - No follow-up mentioned.   Monitor duration   - 6-week | Primary   - Global QoL (questions 29 and 30 of the EORTC-QLQ-C30);   Secondary   - Symptom distress (ESAS), - Symptom complexity (predefined threshold) - Function (KPS, EORTC-QLQ-C30) - Nutrition (symptom appetite, nutritional intake, and weight loss) - Communication (unvalidated scale) | - E-MOSAIC improved symptom distress (P=0.003) - Better symptom management (P=0.06), communication, and coping; no significant effect on G-QoL (P=0.1) |
| Maguire [36]  2015   - UK - Mix method study - Clinical setting | Pop: N=16  Age: (Mean=63.6)  Gender:   - Male N=5, 31%   Type:   - Cancer patients receiving thoracic radiation therapy.   On treatment/ Scheduled for treatment;  28.1% | **Mobile phone-based**   - Mobile phone-based ASyMS-R - Daily e-proms (MSAS-SF, RSC-Activity) - Risk model for alerts - Automated self-care advice   **Symptom monitored**  **NA** | - Clinician leading. - Patient symptom input - Real-time data to server - Risk analysis - Self-care advice to patient + alerts to clinicians - Clinician response   Monitor duration   - Daily (at home, 9 AM-5 PM) - For the duration of the radiotherapy treatment and one month post-treatment | Primary Outcome   - Feasibility and acceptability of ASyMS-R   Secondary Outcome   - Changes in anxiety (STAI-Y) - Self-care self-efficacy (SUPPH-29) - Well-being/quality of life (FACT-L) - Physical symptom distress (ESAS) | - Clinical improvements in anxiety, drowsiness, and self-efficacy in self-care were observed. - System perceived as positive and effective based on qualitative interviews. |
| Yount [37]  2014   - USA - RCT   Outpatient clinic | Pop: N=253  Age:   - IG (Mean=61) - CG (Mean=60.2)   Gender:   - IG (Male N=57, 22%) - CG (Male N=68, 26%)   Type   - Stages III or IV LC   On treatment;  61% | **Telephone-based**   - Weekly symptom telemonitoring via IVR - Automated alerts to nurses for MR group   Longitudinal symptom graphs for MR group at clinic visits  **Symptom monitored**   - Cough, dyspnea, chest pain, pain, fatigue, appetite loss, weight loss, nausea/vomiting, HRQL satisfaction, general cancer symptoms, side‑effect bother - Emotional distress | - Nurse leading - Patients call weekly via IVR - Symptom scores recorded - MR group: alerts emailed to nurses for significant symptoms - Nurse follow-up within 1 day - Graphs provided at visits (MR only); MA group: monitoring without reporting   Monitor duration   - Weekly for 12 weeks | Primary Outcome   - Symptom burden (SDS)   Secondary Outcome   - HRQL (FACT-G) - Treatment satisfaction (FACIT-TS-PS) - Symptom management barriers (SMBQ) - Self-efficacy (custom 27-item measure)   Exploratory: Medical utilization, clinical activity. | - No significant reduction in symptom burden with symptom monitoring and reporting versus monitoring alone in lung cancer patients. - At week 12, treatment satisfaction was higher in MA than MR patients (P < 0.012, P < 0.027). - Adherence to weekly calls was good (82%) and patient satisfaction was high. |

Appendix 2. Characteristics of Included Studies (Continued)

| **Author**  **Year**  **Country**  **Study Type Setting** | **Patient/ Treatment Characteristics**  **Treatment status**  **Adherence rate** | **System Components**  **&**  **Symptom monitored** | **System flow**  **Monitoring duration** | **Outcome Measures/Scales** | **Main findings** |
| --- | --- | --- | --- | --- | --- |
| Berry [38]  2014   - USA - RCT   Cancer hospital | Pop: N=517  Age:   - IG (Mean=55) - CG (Mean=59)   Gender:   - IG (Male N=123, 23%) - CG (Male N=142, 27%)   Type:   - All stages and types of cancer;   Scheduled for treatment;  68% | **Web-based**   - Web-based ESRA-C with SxQOL self-monitoring - Tailored self-care education - Communication coaching for patients   Clinician summaries of patient-reported SxQOL  **Symptom monitored**  **NA** | - Patients report SxQOL via ESRA-C - Clinicians receive summaries - Patients access tracking/education/coaching - Audio-recorded visit at 6 weeks coded for SxQOL discussions   Monitor duration   - Optional access anytime, with key assessments before treatment (T1) and within 24 hours before a clinic visit (T2, ~6 weeks post-treatment start) | Primary Outcome   - Symptom distress (SDS-15) | - Median report index of coached statements: Control Group: 0.25; Intervention Group: 0.31; p-value: 0.008. - Increased reports of fatigue, pain, and physical function issues in the intervention group (all p < .05). - No difference in clinicians' verbalized responses between groups; patients' verbal reports did not mediate final SD outcomes (p = .41). |
| Chih[39] 2013,   - USA - Pooled analysis - Outpatient oncology clinics | Pop: N=235 (LC=64)  Age: (Mean=63)  Gender:   - Male N=104, 44%   Type:   - Breast cancer, prostate cancer, and LC - Stage IIIA, IIIB, or IV   On treatment;  Not specified | **Internet-based**   - Comprehensive Health Enhancement Support System (CHESS): tracking patients status. - Clinician Report (CR): delivers alerts to clinicians. - Caregivers receive immediate support from clinicians via ePRO systems.   **Symptom monitored**  **NA** | - Clinician leading - Caregivers/patients complete Check-in every 7 days via CHESS - CR group: clinicians receive e-mail alerts for ESAS scores ≥7 or 2 days before visits; - CHESS-Only group: no clinician communication   Monitor duration   - Weekly report - Up to 24 months | - Caregiver preparedness (subscale of Family Care Inventory); - Caregiver physical burden (subscale of the Caregiver Burden Inventory); - Caregiver negative mood (subscale of the Shortened Version Profile of Mood States) | - CHESS + CR group reported less negative mood at 6 months (p=0.009) and 12 months (p=0.004); - No significant differences in preparedness (p=0.52) or physical burden (p=0.86) |
| Cox [40]  2011   - US - Mixed method study   Hospices | Pop: N=13 (clinicians only)  No patient participation | **Phone-based**   - Completion of PROM - Data synthesis and instant advice   Automated alerts sent to the clinicians.  **Symptom monitored**  **NA** | - Clinicians leading - Daily ESAS complete - Weekly EQ-5D complete - Overnight data sent to clinicians (Daily) - Real-time alerts sent to the clinicians - Monitor duration not reported | - The acceptability of intervention program (Qualitative interview) | - Potential benefits (empowerment, accurate data recording) - Concerns (age, rapid deterioration, lack of face-to-face contact, standardized tool should not replace clinical judgment) |
| Basch [41]  2007   - USA - Feasibility study,   Outpatient clinic | Pop: N=107 (LC=100)  Age: (Mean=62.0)  Gender:   - Male N=51, 47%   Type:   - LC patients receiving cytotoxic chemotherapy   On treatment/ Scheduled for treatment;  86% | **Internet-based**  Symptom Tracking and Reporting (STAR)  **Symptom monitored**   - Anorexia, constipation, diarrhea, fatigue, nausea, pain, vomiting, cough, dyspnea | - Nurse leading. - Computer-teaching session at enrollment - Patients self-report via clinic computers (optional home access) - Traditional contact (e.g., phone) - An automated alert is sent to nurses and an email is dispatched to clinicians when a predefined symptom severity threshold is reached.   Monitor duration   - Report each visit - Up to 71 weeks | Primary Outcome   - Feasibility (adherence rate)   Secondary Outcome   - Patient satisfaction (questionnaire) - Nurse survey (questionnaire) | - 78% adherence rate over 42 weeks - 90% patient satisfaction, - 15% home reporting, 179 toxicity alerts (e.g., pain, dyspnea), - Nurses found reports useful but rarely discussed due to time constraints |

***Abbreviations:***

NSCLC: Non-small cell lung cancer; MDASI-LC: MD Anderson Symptom Inventory for Lung Cancer; SUS: System Usability Scale; DCC: data collection coordinator; ESAS: Edmonton Symptom Assessment Scale; TIQ: Therapy Impact Questionnaire; DT: Distress Thermometer; PRO-CTCAE: Patient-Reported Outcomes version of the Common Terminology Criteria for Adverse Events; ESAS: Edmonton Symptom Assessment System; PCQ: Physician Compassion Questionnaire; PPQ: Physician Professionalism Questionnaire; GCRS: Global Consultation Rating Scale; JPP: Jefferson Patient Perception of Physician Empathy; PHQ8: Patient Health Quesionnaire-8; GAD-7: Generalized Anxiety Disorder-7; TIMP: Trust in Medical Profession; PREM: Patient Reported Experience Measure; PGHD: Patient-generated health data; PHQ4: Patient Health Questionnaire-4; SIQOL: Single-Item QOL Scale; STAI-Y: State-Trait Anxiety Inventory Form Y; SUPPH-29: Strategies Used by Patients to Promote Health; FACT-L: Functional Assessment of Cancer Therapy-Lung Cancer; SDS: Symptom Distress Scale; FACT-G: Functional Assessment of Cancer Therapy - General; FACIT-TS-PS: Functional Assessment of Chronic Illness Therapy-Treatment Satisfaction-Patient Satisfaction; SMBQ: The Symptom Management Barriers Questionnaire; IPOS: Integrated Palliative care Outcome Scale; CAUQ: Computer System Usability Questionnaire

**References**

1. Stover AM, Deal AM, Medley CJ, Weiner AA, Novak L, Gentry AL, Hoch C, Weiss J, Pecot CV, Lee CB, O’Leary MC, Shrestha S, Chen H, Patel SA, Mody GN. Feasibility, Acceptability, and Utility of Remote Patient-Reported Outcomes Monitoring in Patients With Lung Cancer: A Moovcare© Study. Clinical Lung Cancer 2025 Aug;S1525730425001603. doi: 10.1016/j.cllc.2025.07.016

2. Sewell M, Boerner T, Harrington C, Hsu M, Tan KS, Carr RA, Jones S, Zocco D, Adusumilli PS, Bains MS, Bott MJ, Downey RJ, Huang J, Isbell JM, Park BJ, Rocco G, Rusch VW, Sihag S, Jones DR, Cracchiolo J, Molena D. Remote Symptom Monitoring in Thoracic Surgery Patients After Discharge. Annals of Surgery 2025 June;281(6):1063–1069. doi: 10.1097/SLA.0000000000006619

3. Pongiglione B, Cucciniello M, Petracca F, Ciani O, Novello S, Migliorino M, Pedrazzoli P, Agustoni F, Lo Russo G, Tarricone R, Capelletto E. A mobile supportive care app for patients with metastatic lung cancer: the Lung Cancer App (LuCApp) randomized controlled trial. Support Care Cancer 2025 July;33(7):641. doi: 10.1007/s00520-025-09682-5

4. Nuamek T, Kwateng PAN, Payne A, Abdulwahid D, Barker C, Banfill K, Bayman N, Bowen Jones S, Chan C, Gurumurthy G, Harris M, Horne A, King J, Pemberton L, Sheikh HY, Thomson D, Woolf D, Yorke J, Price J, Faivre-Finn C. Integrating Electronic Patient-Reported Outcome Measures (ePROMs) into Personalised Follow-up for Patients after Radiotherapy. A Feasibility Study. Technical Innovations & Patient Support in Radiation Oncology 2025 Sept;35:100333. doi: 10.1016/j.tipsro.2025.100333

5. Jing P, Liang Y, Tan Z, Yan X, Lei J, Ni Y, Zhu X, Qiu C, Wang J, Ge P, Zhang Y, Wang L, Zhao N, Zhang Y, Wang J, Wang Y, Zheng C, Shao Q, Zhang H, Yang Z, Li H, Fan J, Liu S, Kyriacou K, Shang L, Gu Z. Physiological and psychological symptom management based on electronic patient-reported outcomes: the TD-WELLBEING randomized clinical trial. Br J Cancer 2025 Oct 19;133(7):937–944. doi: 10.1038/s41416-025-03110-5

6. Lv C, Lu F, Zhou X, Li X, Yu W, Zhang C, Chen K, Du S, Han C, Wang J, Wang Y, Li S, Wang L, Liu Y, Zhang S, Huang M, Song D, Zhao D, Liu B, Wang Y, Cui X, Zhou Z, Yan S, Wu N. Efficacy of a smartphone application assisting home-based rehabilitation and symptom management for patients with lung cancer undergoing video-assisted thoracoscopic lobectomy: a prospective, single-blinded, randomised control trial (POPPER study). International Journal of Surgery 2025 Jan;111(1):597. doi: 10.1097/JS9.0000000000001845

7. Blakely LJ, Oskar S, Kudel I, Roush A, Shamsi Z, Perry T, Christianson A, Smith B, Burke T. Real-world ePRO use and clinical outcomes using electronic patient-reported symptom monitoring for patients with advanced non-small-cell lung cancer receiving first-line pembrolizumab. J Comp Eff Res 2025 Feb;14(2):e240122. doi: 10.57264/cer-2024-0122

8. Yang GM, Ke Y, Ng XH, Neo PSH, Cheung YB. Proactive symptom monitoring to initiate timely palliative care for patients with advanced cancer: a randomized controlled trial. Support Care Cancer 2025 Mar;33(3):249. doi: 10.1007/s00520-025-09311-1

9. Yu H, Lei C, Wei X, Wang Y, Xu W, Tang L, Dai W, Liao J, Pu Y, Gong R, Su X, Yu Q, Zhang J, Zhang L, Huang Y, Zhuang X, Bai J, Wang Z, Li Q, Shi Q. Electronic symptom monitoring after lung cancer surgery: establishing a core set of patient-reported outcomes for surgical oncology care in a longitudinal cohort study. International Journal of Surgery 2024 Oct;110(10):6591. doi: 10.1097/JS9.0000000000001855

10. Mooney K, Gullatte M, Iacob E, Alekhina N, Nicholson B, Sloss EA, Lloyd J, Moraitis AM, Donaldson G. Essential Components of an Electronic Patient-Reported Symptom Monitoring and Management System: A Randomized Clinical Trial. JAMA Netw Open 2024 Sept 13;7(9):e2433153. doi: 10.1001/jamanetworkopen.2024.33153

11. Dai W, Wang Y, Liao J, Wei X, Dai Z, Xu W, Liu Y, Wang XS, Pompili C, Yu H, Pu Y, Zhao Y, Cao B, Wang Q, Feng W, Zhang Y, Liu F, Deng Y, Zhou J, Li J, Xie S, Xiang R, Wang X, Tian B, Yang X, Hu B, Liu X, Xie T, Yang X, Zhuang X, Qiao G, Li Q, Shi Q. Electronic Patient-Reported Outcome–Based Symptom Management Versus Usual Care After Lung Cancer Surgery: Long-Term Results of a Multicenter, Randomized, Controlled Trial. JCO 2024 Apr 4;JCO.23.01854. doi: 10.1200/JCO.23.01854

12. Friis RB, Pappot H, Hjollund NH, McCulloch T, Holt MI, Persson GF, Wedervang K, Clausen MM, Wahlstrøm S, Hansen KH, Rasmussen TR, Dalton SO, Jakobsen E, Linnet H, Skuladottir H, The Danish Lung Cancer Group. Remote Symptom Monitoring of Patients With Advanced Lung Cancer (The ProWide Study): A Randomized Controlled Trial. JCO Oncol Pract 2024 Dec 10;OP-24-00562. doi: 10.1200/OP-24-00562

13. Boisson-Walsh A, Cox C, O’Leary M, Shrestha S, Carr P, Gentry AL, Hill L, Newsome B, Long J, Haithcock B, Stover AM, Basch E, Leeman J, Mody GN. A Qualitative Study of Electronic Patient-Reported Outcome Symptom Monitoring After Thoracic Surgery. Journal of Surgical Research 2024 Nov;303:744–755. doi: 10.1016/j.jss.2024.09.051

14. Arriola E, Jaal J, Edvardsen A, Silvoniemi M, Araújo A, Vikström A, Zairi E, Rodriguez-Mues MC, Roccato M, Schneider S, Ammann J. Feasibility and User Experience of Digital Patient Monitoring for Real-World Patients With Lung or Breast Cancer. The Oncologist 2024 Apr 4;29(4):e561–e569. doi: 10.1093/oncolo/oyad289

15. Payne A, Horne A, Bayman N, Blackhall F, Bostock L, Chan C, Coote J, Eaton M, Fenemore J, Gomes F, Halkyard E, Harris M, Lindsay C, McEntee D, Neal H, Pemberton L, Sheikh H, Woolf D, Price J, Yorke J, Faivre-Finn C. Patient and clinician-reported experiences of using electronic patient reported outcome measures (ePROMs) as part of routine cancer care. Journal of Patient-Reported Outcomes 2023 May 4;7(1):42. doi: 10.1186/s41687-023-00544-4

16. Patt DA, Patel AM, Bhardwaj A, Hudson KE, Christman A, Amondikar N, Escudier SM, Townsend S, Books H, Basch E. Impact of Remote Symptom Monitoring With Electronic Patient-Reported Outcomes on Hospitalization, Survival, and Cost in Community Oncology Practice: The Texas Two-Step Study. JCO Clinical Cancer Informatics 2023 Sept;(7):e2300182. doi: 10.1200/CCI.23.00182

17. Parikh RB, Schriver E, Ferrell WJ, Wakim J, Williamson J, Khan N, Kopinsky M, Balachandran M, Gabriel PE, Schuchter LM, Patel MS, Shulman LN, Manz CR. Remote Patient-Reported Outcomes and Activity Monitoring to Improve Patient-Clinician Communication Regarding Symptoms and Functional Status: A Randomized Controlled Trial. JCO Oncol Pract 2023 Dec;19(12):1143–1151. PMID:37816198

18. Ma TM, Yang T, Philipson R, Kishan AU, Lee P, Raldow AC. Web-Based Symptom Monitoring With Patient-Reported Outcomes During Definitive Radiation Therapy With Chemotherapy (SYMPATHY): A Prospective Single-Center Phase 1 Study. Advances in Radiation Oncology Elsevier; 2023 May 1;8(3). PMID:36532603

19. Lee M, Kang D, Kang E, Kim S, Kim Y, Ahn JS, Park S, Lee Y-Y, Oh D, Noh JM, Cho J. Efficacy of the PRO-CTCAE mobile application for improving patient participation in symptom management during cancer treatment: a randomized controlled trial. Support Care Cancer 2023 June;31(6):321. doi: 10.1007/s00520-023-07779-3

20. Schougaard LMV, Friis RB, Grytnes R, Grove BE, Hjollund NH, Pappot H, Skuladottir H, Mejdahl CT. Exploring the Nurses’ Perspective on Using Remote Electronic Symptom Monitoring in Clinical Decision-Making Among Patients With Metastatic Lung Cancer. Seminars in Oncology Nursing 2023 Dec;39(6):151517. doi: 10.1016/j.soncn.2023.151517

21. Wujcik D, Dudley WN, Dudley M, Gupta V, Brant J. Electronic Patient Symptom Management Program to Support Patients Receiving Cancer Treatment at Home During the COVID-19 Pandemic. Value in Health Elsevier; 2022 June 1;25(6):931–936. PMID:35339378

22. Dai W, Feng W, Zhang Y, Wang XS, Liu Y, Pompili C, Xu W, Xie S, Wang Y, Liao J, Wei X, Xiang R, Hu B, Tian B, Yang X, Wang X, Xiao P, Lai Q, Wang X, Cao B, Wang Q, Liu F, Liu X, Xie T, Yang X, Zhuang X, Wu Z, Che G, Li Q, Shi Q. Patient-Reported Outcome-Based Symptom Management Versus Usual Care After Lung Cancer Surgery: A Multicenter Randomized Controlled Trial. JCO Wolters Kluwer; 2022 Mar 20;40(9):988–996. doi: 10.1200/JCO.21.01344

23. Girgis A, Bamgboje-Ayodele A, Rincones O, Vinod SK, Avery S, Descallar J, Smith A ‘Ben,’ Arnold B, Arnold A, Bray V, Durcinoska I, Rankin NM, the PROMPT-Care Implementation Authorship Group, Chang CF, Eifler B, Elliott S, Hardy C, Ivimey B, Jansens W, Kaadan N, Koh E-S, Livio N, Lozenkovski S, McErlean G, Nasser E, Ryan N, Smeal T, Thomas T, Tran T, Wiltshire J, Delaney GP. Stepping into the real world: a mixed-methods evaluation of the implementation of electronic patient reported outcomes in routine lung cancer care. J Patient Rep Outcomes 2022 Dec;6(1):70. doi: 10.1186/s41687-022-00475-6

24. Zhang L, Zhang X, Shen L, Zhu D, Ma S, Cong L. Efficiency of Electronic Health Record Assessment of Patient-Reported Outcomes After Cancer Immunotherapy: A Randomized Clinical Trial. JAMA Network Open 2022;5(3):e224427. PMID:35357459

25. Mody GN, Stover AM, Wang M, King-Kallimanis BL, Jansen J, Henson S, Chung AE, Jonsson M, Bennett A, Smith AB, Wood WA, Deal A, Ginos B, Dueck AC, Schrag D, Basch E. Electronic patient-reported outcomes monitoring during lung cancer chemotherapy: A nested cohort within the PRO-TECT pragmatic trial (AFT-39). Lung Cancer 2021 Dec;162:1–8. doi: 10.1016/j.lungcan.2021.09.020

26. Cheng X, Yang Y, Shentu Y, Ding Z, Zhou Q, Tan Q, Luo Q. Remote monitoring of patient recovery following lung cancer surgery: a messenger application approach. J Thorac Dis 2021 Feb;13(2):1162–1171. doi: 10.21037/jtd-21-27

27. Zylla DM, Gilmore GE, Steele GL, Eklund JP, Wood CM, Stover AM, Shapiro AC. Collection of electronic patient-reported symptoms in patients with advanced cancer using Epic MyChart surveys. Support Care Cancer 2020 July 1;28(7):3153–3163. doi: 10.1007/s00520-019-05109-0

28. Iivanainen S, Alanko T, Vihinen P, Konkola T, Ekstrom J, Virtanen H, Koivunen J. Follow-Up of Cancer Patients Receiving Anti-PD-(L)1 Therapy Using an Electronic Patient-Reported Outcomes Tool (KISS): Prospective Feasibility Cohort Study. JMIR Form Res 2020 Oct 28;4(10):e17898. doi: 10.2196/17898

29. Friis RB, Hjollund NH, Mejdahl CT, Pappot H, Skuladottir H. Electronic symptom monitoring in patients with metastatic lung cancer: a feasibility study. BMJ Open 2020 June;10(6):e035673. doi: 10.1136/bmjopen-2019-035673

30. Schmalz O, Jacob C, Ammann J, Liss B, Iivanainen S, Kammermann M, Koivunen J, Klein A, Popescu RA. Digital Monitoring and Management of Patients With Advanced or Metastatic Non-Small Cell Lung Cancer Treated With Cancer Immunotherapy and Its Impact on Quality of Clinical Care: Interview and Survey Study Among Health Care Professionals and Patients. J Med Internet Res 2020 Dec 21;22(12):e18655. doi: 10.2196/18655

31. Gustafson DH, DuBenske LL, Atwood AK, Chih M-Y, Johnson RA, McTavish F, Quanbeck A, Brown RL, Cleary JF, Shah D. Reducing Symptom Distress in Patients With Advanced Cancer Using an e-Alert System for Caregivers: Pooled Analysis of Two Randomized Clinical Trials. J Med Internet Res 2017 Nov 14;19(11):e354. doi: 10.2196/jmir.7466

32. Mooney KH, Beck SL, Wong B, Dunson W, Wujcik D, Whisenant M, Donaldson G. Automated home monitoring and management of patient‐reported symptoms during chemotherapy: results of the symptom care at home. Cancer Medicine 2017 Mar;6(3):537–546. doi: 10.1002/cam4.1002

33. Denis F, Lethrosne C, Pourel N, Molinier O, Pointreau Y, Domont J, Bourgeois H, Senellart H, Trémolières P, Lizée T, Bennouna J, Urban T, El Khouri C, Charron A, Septans A-L, Balavoine M, Landry S, Solal-Céligny P, Letellier C. Randomized Trial Comparing a Web-Mediated Follow-up With Routine Surveillance in Lung Cancer Patients. JNCI: Journal of the National Cancer Institute 2017 Sept 1;109(9). doi: 10.1093/jnci/djx029

34. Basch E, Deal AM, Kris MG, Scher HI, Hudis CA, Sabbatini P, Rogak L, Bennett AV, Dueck AC, Atkinson TM, Chou JF, Dulko D, Sit L, Barz A, Novotny P, Fruscione M, Sloan JA, Schrag D. Symptom Monitoring With Patient-Reported Outcomes During Routine Cancer Treatment: A Randomized Controlled Trial. JCO 2016 Feb 20;34(6):557–565. doi: 10.1200/JCO.2015.63.0830

35. Strasser F, Blum D, Von Moos R, Cathomas R, Ribi K, Aebi S, Betticher D, Hayoz S, Klingbiel D, Brauchli P, Haefner M, Mauri S, Kaasa S, Koeberle D. The effect of real-time electronic monitoring of patient-reported symptoms and clinical syndromes in outpatient workflow of medical oncologists: E-MO AIC, a multicenter cluster-randomized phase III study (SAKK 95/06). Annals of Oncology 2016 Feb;27(2):324–332. doi: 10.1093/annonc/mdv576

36. Maguire R, Ream E, Richardson A, Connaghan J, Johnston B, Kotronoulas G, Pedersen V, McPhelim J, Pattison N, Smith A, Webster L, Taylor A, Kearney N. Development of a Novel Remote Patient Monitoring System: The Advanced Symptom Management System for Radiotherapy to Improve the Symptom Experience of Patients With Lung Cancer Receiving Radiotherapy. Cancer Nursing 2015 Mar;38(2):E37–E47. doi: 10.1097/NCC.0000000000000150

37. Yount SE, Rothrock N, Bass M, Beaumont JL, Pach D, Lad T, Patel J, Corona M, Weiland R, Del Ciello K, Cella D. A Randomized Trial of Weekly Symptom Telemonitoring in Advanced Lung Cancer. Journal of Pain and Symptom Management 2014 June;47(6):973–989. doi: 10.1016/j.jpainsymman.2013.07.013

38. Berry DL, Hong F, Halpenny B, Partridge A, Fox E, Fann JR, Wolpin S, Lober WB, Bush N, Parvathaneni U, Amtmann D, Ford R. The electronic self report assessment and intervention for cancer: promoting patient verbal reporting of symptom and quality of life issues in a randomized controlled trial. BMC Cancer 2014 July 12;14(1):513. doi: 10.1186/1471-2407-14-513

39. Chih M-Y, DuBenske LL, Hawkins RP, Brown RL, Dinauer SK, Cleary JF, Gustafson DH. Communicating advanced cancer patients’ symptoms via the Internet: A pooled analysis of two randomized trials examining caregiver preparedness, physical burden, and negative mood. Palliat Med 2013 June;27(6):533–543. doi: 10.1177/0269216312457213

40. Cox A, Illsley M, Knibb W, Lucas C, O’Driscoll M, Potter C, Flowerday A, Faithfull S. The acceptability of e-technology to monitor and assess patient symptoms following palliative radiotherapy for lung cancer. Palliat Med 2011 Oct;25(7):675–681. doi: 10.1177/0269216311399489

41. Basch E, Iasonos A, Barz A, Culkin A, Kris MG, Artz D, Fearn P, Speakman J, Farquhar R, Scher HI, McCabe M, Schrag D. Long-Term Toxicity Monitoring via Electronic Patient-Reported Outcomes in Patients Receiving Chemotherapy. JCO 2007 Dec 1;25(34):5374–5380. doi: 10.1200/JCO.2007.11.2243
