# Supplementary material for: The Current Landscape of Remote Digital Symptom Monitoring for Patients With Lung Cancer: Scoping Review
Source: J Med Internet Res. 2026 Mar 24;28:e83666. doi: 10.2196/83666 (PMC13012230; doi:10.2196/83666)
Supplement: Multimedia Appendix 3 [file jmir-v28-e83666-s003.docx]

| Domain Measured | Research Team | PROM | Purpose | |
| --- | --- | --- | --- | --- |
|  |  |  | Study outcome measures | rSMS component |
| Symptom burden | Yang et al., 2025[1] | IPOS |  | ☑ |
|  | Stover et al., 2025[2] | Self-developed tool |  | ☑ |
|  | Sewell et al., 2025[3] | Self-developed tool |  | ☑ |
|  | Blakely et al., 2025[4] | Self-developed tool |  | ☑ |
|  | Yu et al., 2024[5] | Self-developed tool |  | ☑ |
|  | Mooney et al., 2024[6] | Self-developed tool |  | ☑ |
|  | Arriola et al., 2024[7] | Self-developed tool |  | ☑ |
|  | Schougaard et al., 2023[8] | Self-developed tool |  | ☑ |
|  | Denis et al., 2017[9] | Self-developed tool |  | ☑ |
|  | Basch et al., 2007[10] | Self-developed tool |  | ☑ |
|  | Cheng et al., 2021[11] | Numeric rating score |  | ☑ |
|  | Mooney et al., 2017[12] | Numeric rating score |  | ☑ |
|  | Friis et al., 2024[13] | EORTC Item Library (adapted) |  | ☑ |
|  | Friis et al., 2020[14] | EORTC Item Library (adapted) |  | ☑ |
|  | Pongiglione et al., 2025[15] | CTCAE (adapted) |  | ☑ |
|  | Parikh et al., 2023[16] | CTCAE |  | ☑ |
|  | Nuamek et al., 2025[17] | CTCAE v5.0 (adapted) |  | ☑ |
|  | Payne et al., 2023[18] | CTCAE v5.0 (adapted) |  | ☑ |
|  | Zhang et al., 2022[19] | CTCAE v5.0 (adapted) |  | ☑ |
|  | Yu et al., 2025[20] | LC13 |  | ☑ |
|  | Yu et al., 2025[20] | EORTC-QLQ-C30 |  | ☑ |
|  | Yu et al., 2025[20] | MDASI-LC | ☑ |  |
|  | Jing et al., 2025[21] | MDASI-LC |  | ☑ |
|  | Dai et al., 2024[22] | MDASI-LC |  | ☑ |
|  | Dai et al.,2022[23] | MDASI-LC |  | ☑ |
|  | Ma et al., 2023[24] | PRO-CTCAE |  | ☑ |
|  | Mody et al., 2021[25] | PRO-CTCAE |  | ☑ |
|  | Schmalz et al., 2020[26] | PRO-CTCAE |  | ☑ |
|  | Wujcik et al., 2022[27] | PRO-CTCAE (adapted) |  | ☑ |
|  | Lee et al., 2023[28] | PRO-CTCAE-K |  | ☑ |
|  | Girgis et al., 2022[29] | ESAS |  | ☑ |
|  | Strasser et al., 2016[30] | ESAS | ☑ | ☑ |
|  | Maguire et al., 2015[31] | ESAS | ☑ |  |
|  | Cox et al., [32] | ESAS |  | ☑ |
|  | Gustafson et al., 2017[33] | ESAS (adapted) |  | ☑ |
|  | Zylla et al., 2020[34] | PRSM |  | ☑ |
|  | Iivanainen et al., 2020[35] | NCI-CTCAE (adapted) |  | ☑ |
|  | Basch et al., 2016[36] | NCI-CTCAE (adapted) |  | ☑ |
|  | Maguire et al., 2015[31] | RSC-Activity |  | ☑ |
|  | Yount et al., 2014[37] | SDS | ☑ |  |
|  | Berry et al., 2014[38] | SDS-15 | ☑ |  |
|  | Berry et al., 2014[38] | ESRA-C |  | ☑ |
| Health related quality of life | Stover et al., 2025[2] | EORTC-QLQ-C30 | ☑ |  |
|  | Friis et al., 2024[13] | EORTC QLQ-C30 | ☑ |  |
|  | Lee et al., 2023[28] | EORTC QLQ-C30 | ☑ |  |
|  | Mody et al., 2021[25] | EORTC QLQ-C30 | ☑ |  |
|  | Strasser et al., 2016[30] | EORTC QLQ-C30 (adapted) | ☑ |  |
|  | Jing et al., 2025[21] | QLQ-C30 | ☑ |  |
|  | Arriola et al., 2024[7] | QLQ-C30 | ☑ |  |
|  | Zhang et al., 2022[19] | QLQ-C30 | ☑ |  |
|  | Stover et al., 2025[2] | QLQ-LC13 | ☑ |  |
|  | Friis et al., 2024[13] | QLQ-LC13 | ☑ |  |
|  | Pongiglione et al., 2025[15] | FACT-L | ☑ |  |
|  | Maguire et al., 2015[31] | FACT-L | ☑ |  |
|  | Yount et al., 2014[37] | FACT-G | ☑ |  |
|  | Pongiglione et al., 2025[15] | EuroQoL 5D-5L | ☑ |  |
|  | Nuamek et al., 2025[17] | EuroQoL 5D-5L |  | ☑ |
|  | Friis et al., 2024[13] | EuroQol 5D-5L | ☑ |  |
|  | Payne et al., 2023[18] | EuroQol 5D-5L |  | ☑ |
|  | Basch et al., 2016[36] | EuroQol 5D-5L | ☑ |  |
|  | Cox et al., 2011[32] | EuroQol 5D-5L |  | ☑ |
|  | Basch et al,. 2007[10] | EuroQol 5D-5L (adapted) |  | ☑ |
|  | Pongiglione et al., 2025[15] | HADS | ☑ |  |
|  | Friis et al., 2024[13] | EORTC Item Library (adapted) |  | ☑ |
|  | Friis et al., 2024[13] | HADS | ☑ |  |
|  | Ma et al., 2023[24] | PROMIS | ☑ |  |
|  | Dai et al.,2022[23] | SIQOL |  | ☑ |
|  | Maguire et al., 2015[31] | MSAS-SF |  | ☑ |
|  | Yount et al., 2014[37] | FLSI |  | ☑ |
| Psychosocial wellbeing | Girgis et al., 2022[29] | Distress Thermometer |  | ☑ |
|  | Maguire et al., 2015[31] | STAI-Y | ☑ |  |
| Exercise intensity | Yu et al., 2025[20] | 10-point Borg scale of shortness of breath | ☑ |  |
| Sefl-care efficacy | Maguire et al., 2015[31] | SUPPH-29 | ☑ |  |
| Treatment satisfaction | Yount et al., 2014[37] | FACIT-TS-S | ☑ |  |

Abbreviations:

IPOS: Integrated Palliative care Outcome Scale; SIQOL: Single item QoL scale; MDASI-LC: MD Anderson Symptom Inventory-Lung Cancer module; PRO-CTCAE: National Cancer Institute’s Patient-Reported Outcomes version of the Common Terminology Criteria for Adverse Events; PRSM: Patient-Reported Symptom Monitoring; NCI-CTCAE: National Cancer Institute Common Terminology Criteria for Adverse Events; MSAS-SF: Memorial Symptom Assessment Scale – Short Form; STAI-Y: State-Trait Anxiety Inventory Form Y; SUPPH-29: Strategies Used by Patients to Promote Health; RSC-Activity: Rotterdam Symptom Checklist – Activity Subscale; FLSI: Functional Assessment of Cancer Therapy [FACT] e Lung Symptom Index; SDS: Symptom Distress Scale; FACT-G: FACT-General; FACIT-TS-S: Functional Assessment of Chronic Illness Therapy-Treatment Satisfaction-Patient Satisfaction; ESRA-C: electronic self report assessment for cancer; EORTC: European Organisation for Research and Treatment of Cancer; EORTC-QLQ-C30European Organisation for Research and Treatment of Cancer Quality of Life Questionnaire Core 30; PRO-CTCAE-K: Patient-Reported Outcomes version of the Common Terminology Criteria for Adverse Events – Korean; ESAS: Edmonton Symptom Assessment System; HADS: Hospital Anxiety and Depression Scale; PROMIS: Patient-Reported Outcomes Measurement Information System

References:

1. Yang GM, Ke Y, Ng XH, Neo PSH, Cheung YB. Proactive symptom monitoring to initiate timely palliative care for patients with advanced cancer: a randomized controlled trial. Support Care Cancer 2025 Mar;33(3):249. doi: 10.1007/s00520-025-09311-1

2. Stover AM, Deal AM, Medley CJ, Weiner AA, Novak L, Gentry AL, Hoch C, Weiss J, Pecot CV, Lee CB, O’Leary MC, Shrestha S, Chen H, Patel SA, Mody GN. Feasibility, Acceptability, and Utility of Remote Patient-Reported Outcomes Monitoring in Patients With Lung Cancer: A Moovcare© Study. Clinical Lung Cancer 2025 Aug;S1525730425001603. doi: 10.1016/j.cllc.2025.07.016

3. Sewell M, Boerner T, Harrington C, Hsu M, Tan KS, Carr RA, Jones S, Zocco D, Adusumilli PS, Bains MS, Bott MJ, Downey RJ, Huang J, Isbell JM, Park BJ, Rocco G, Rusch VW, Sihag S, Jones DR, Cracchiolo J, Molena D. Remote Symptom Monitoring in Thoracic Surgery Patients After Discharge. Annals of Surgery 2025 June;281(6):1063–1069. doi: 10.1097/SLA.0000000000006619

4. Blakely LJ, Oskar S, Kudel I, Roush A, Shamsi Z, Perry T, Christianson A, Smith B, Burke T. Real-world ePRO use and clinical outcomes using electronic patient-reported symptom monitoring for patients with advanced non-small-cell lung cancer receiving first-line pembrolizumab. J Comp Eff Res 2025 Feb;14(2):e240122. doi: 10.57264/cer-2024-0122

5. Yu H, Lei C, Wei X, Wang Y, Xu W, Tang L, Dai W, Liao J, Pu Y, Gong R, Su X, Yu Q, Zhang J, Zhang L, Huang Y, Zhuang X, Bai J, Wang Z, Li Q, Shi Q. Electronic symptom monitoring after lung cancer surgery: establishing a core set of patient-reported outcomes for surgical oncology care in a longitudinal cohort study. International Journal of Surgery 2024 Oct;110(10):6591–6600. doi: 10.1097/JS9.0000000000001855

6. Mooney K, Gullatte M, Iacob E, Alekhina N, Nicholson B, Sloss EA, Lloyd J, Moraitis AM, Donaldson G. Essential Components of an Electronic Patient-Reported Symptom Monitoring and Management System: A Randomized Clinical Trial. JAMA Netw Open 2024 Sept 13;7(9):e2433153. doi: 10.1001/jamanetworkopen.2024.33153

7. Arriola E, Jaal J, Edvardsen A, Silvoniemi M, Araújo A, Vikström A, Zairi E, Rodriguez-Mues MC, Roccato M, Schneider S, Ammann J. Feasibility and User Experience of Digital Patient Monitoring for Real-World Patients With Lung or Breast Cancer. The Oncologist 2024 Apr 4;29(4):e561–e569. doi: 10.1093/oncolo/oyad289

8. Schougaard LMV, Friis RB, Grytnes R, Grove BE, Hjollund NH, Pappot H, Skuladottir H, Mejdahl CT. Exploring the Nurses’ Perspective on Using Remote Electronic Symptom Monitoring in Clinical Decision-Making Among Patients With Metastatic Lung Cancer. Seminars in Oncology Nursing 2023 Dec;39(6):151517. doi: 10.1016/j.soncn.2023.151517

9. Denis F, Lethrosne C, Pourel N, Molinier O, Pointreau Y, Domont J, Bourgeois H, Senellart H, Trémolières P, Lizée T, Bennouna J, Urban T, El Khouri C, Charron A, Septans A-L, Balavoine M, Landry S, Solal-Céligny P, Letellier C. Randomized Trial Comparing a Web-Mediated Follow-up With Routine Surveillance in Lung Cancer Patients. JNCI: Journal of the National Cancer Institute 2017 Sept 1;109(9). doi: 10.1093/jnci/djx029

10. Basch E, Iasonos A, Barz A, Culkin A, Kris MG, Artz D, Fearn P, Speakman J, Farquhar R, Scher HI, McCabe M, Schrag D. Long-Term Toxicity Monitoring via Electronic Patient-Reported Outcomes in Patients Receiving Chemotherapy. JCO 2007 Dec 1;25(34):5374–5380. doi: 10.1200/JCO.2007.11.2243

11. Cheng X, Yang Y, Shentu Y, Ding Z, Zhou Q, Tan Q, Luo Q. Remote monitoring of patient recovery following lung cancer surgery: a messenger application approach. J Thorac Dis 2021 Feb;13(2):1162–1171. doi: 10.21037/jtd-21-27

12. Mooney KH, Beck SL, Wong B, Dunson W, Wujcik D, Whisenant M, Donaldson G. Automated home monitoring and management of patient‐reported symptoms during chemotherapy: results of the symptom care at home. Cancer Medicine 2017 Mar;6(3):537–546. doi: 10.1002/cam4.1002

13. Friis RB, Pappot H, Hjollund NH, McCulloch T, Holt MI, Persson GF, Wedervang K, Clausen MM, Wahlstrøm S, Hansen KH, Rasmussen TR, Dalton SO, Jakobsen E, Linnet H, Skuladottir H, The Danish Lung Cancer Group. Remote Symptom Monitoring of Patients With Advanced Lung Cancer (The ProWide Study): A Randomized Controlled Trial. JCO Oncol Pract 2024 Dec 10;OP-24-00562. doi: 10.1200/OP-24-00562

14. Friis RB, Hjollund NH, Mejdahl CT, Pappot H, Skuladottir H. Electronic symptom monitoring in patients with metastatic lung cancer: a feasibility study. BMJ Open 2020 June;10(6):e035673. doi: 10.1136/bmjopen-2019-035673

15. Pongiglione B, Cucciniello M, Petracca F, Ciani O, Novello S, Migliorino M, Pedrazzoli P, Agustoni F, Lo Russo G, Tarricone R, Capelletto E. A mobile supportive care app for patients with metastatic lung cancer: the Lung Cancer App (LuCApp) randomized controlled trial. Support Care Cancer 2025 July;33(7):641. doi: 10.1007/s00520-025-09682-5

16. Parikh RB, Schriver E, Ferrell WJ, Wakim J, Williamson J, Khan N, Kopinsky M, Balachandran M, Gabriel PE, Schuchter LM, Patel MS, Shulman LN, Manz CR. Remote Patient-Reported Outcomes and Activity Monitoring to Improve Patient-Clinician Communication Regarding Symptoms and Functional Status: A Randomized Controlled Trial. JCO Oncol Pract 2023 Dec;19(12):1143–1151. PMID:37816198

17. Nuamek T, Kwateng PAN, Payne A, Abdulwahid D, Barker C, Banfill K, Bayman N, Bowen Jones S, Chan C, Gurumurthy G, Harris M, Horne A, King J, Pemberton L, Sheikh HY, Thomson D, Woolf D, Yorke J, Price J, Faivre-Finn C. Integrating Electronic Patient-Reported Outcome Measures (ePROMs) into Personalised Follow-up for Patients after Radiotherapy. A Feasibility Study. Technical Innovations & Patient Support in Radiation Oncology 2025 Sept;35:100333. doi: 10.1016/j.tipsro.2025.100333

18. Payne A, Horne A, Bayman N, Blackhall F, Bostock L, Chan C, Coote J, Eaton M, Fenemore J, Gomes F, Halkyard E, Harris M, Lindsay C, McEntee D, Neal H, Pemberton L, Sheikh H, Woolf D, Price J, Yorke J, Faivre-Finn C. Patient and clinician-reported experiences of using electronic patient reported outcome measures (ePROMs) as part of routine cancer care. Journal of Patient-Reported Outcomes 2023 May 4;7(1):42. doi: 10.1186/s41687-023-00544-4

19. Zhang L, Zhang X, Shen L, Zhu D, Ma S, Cong L. Efficiency of Electronic Health Record Assessment of Patient-Reported Outcomes After Cancer Immunotherapy: A Randomized Clinical Trial. JAMA Network Open 2022;5(3):e224427. PMID:35357459

20. Lv C, Lu F, Zhou X, Li X, Yu W, Zhang C, Chen K, Du S, Han C, Wang J, Wang Y, Li S, Wang L, Liu Y, Zhang S, Huang M, Song D, Zhao D, Liu B, Wang Y, Cui X, Zhou Z, Yan S, Wu N. Efficacy of a smartphone application assisting home-based rehabilitation and symptom management for patients with lung cancer undergoing video-assisted thoracoscopic lobectomy: a prospective, single-blinded, randomised control trial (POPPER study). International Journal of Surgery 2025 Jan;111(1):597. doi: 10.1097/JS9.0000000000001845

21. Jing P, Liang Y, Tan Z, Yan X, Lei J, Ni Y, Zhu X, Qiu C, Wang J, Ge P, Zhang Y, Wang L, Zhao N, Zhang Y, Wang J, Wang Y, Zheng C, Shao Q, Zhang H, Yang Z, Li H, Fan J, Liu S, Kyriacou K, Shang L, Gu Z. Physiological and psychological symptom management based on electronic patient-reported outcomes: the TD-WELLBEING randomized clinical trial. Br J Cancer 2025 Oct 19;133(7):937–944. doi: 10.1038/s41416-025-03110-5

22. Dai W, Wang Y, Liao J, Wei X, Dai Z, Xu W, Liu Y, Wang XS, Pompili C, Yu H, Pu Y, Zhao Y, Cao B, Wang Q, Feng W, Zhang Y, Liu F, Deng Y, Zhou J, Li J, Xie S, Xiang R, Wang X, Tian B, Yang X, Hu B, Liu X, Xie T, Yang X, Zhuang X, Qiao G, Li Q, Shi Q. Electronic Patient-Reported Outcome–Based Symptom Management Versus Usual Care After Lung Cancer Surgery: Long-Term Results of a Multicenter, Randomized, Controlled Trial. JCO 2024 Apr 4;JCO.23.01854. doi: 10.1200/JCO.23.01854

23. Dai W, Feng W, Zhang Y, Wang XS, Liu Y, Pompili C, Xu W, Xie S, Wang Y, Liao J, Wei X, Xiang R, Hu B, Tian B, Yang X, Wang X, Xiao P, Lai Q, Wang X, Cao B, Wang Q, Liu F, Liu X, Xie T, Yang X, Zhuang X, Wu Z, Che G, Li Q, Shi Q. Patient-Reported Outcome-Based Symptom Management Versus Usual Care After Lung Cancer Surgery: A Multicenter Randomized Controlled Trial. JCO Wolters Kluwer; 2022 Mar 20;40(9):988–996. doi: 10.1200/JCO.21.01344

24. Ma TM, Yang T, Philipson R, Kishan AU, Lee P, Raldow AC. Web-Based Symptom Monitoring With Patient-Reported Outcomes During Definitive Radiation Therapy With Chemotherapy (SYMPATHY): A Prospective Single-Center Phase 1 Study. Advances in Radiation Oncology Elsevier; 2023 May 1;8(3). PMID:36532603

25. Mody GN, Stover AM, Wang M, King-Kallimanis BL, Jansen J, Henson S, Chung AE, Jonsson M, Bennett A, Smith AB, Wood WA, Deal A, Ginos B, Dueck AC, Schrag D, Basch E. Electronic patient-reported outcomes monitoring during lung cancer chemotherapy: A nested cohort within the PRO-TECT pragmatic trial (AFT-39). Lung Cancer 2021 Dec;162:1–8. doi: 10.1016/j.lungcan.2021.09.020

26. Schmalz O, Jacob C, Ammann J, Liss B, Iivanainen S, Kammermann M, Koivunen J, Klein A, Popescu RA. Digital Monitoring and Management of Patients With Advanced or Metastatic Non-Small Cell Lung Cancer Treated With Cancer Immunotherapy and Its Impact on Quality of Clinical Care: Interview and Survey Study Among Health Care Professionals and Patients. J Med Internet Res 2020 Dec 21;22(12):e18655. doi: 10.2196/18655

27. Wujcik D, Dudley WN, Dudley M, Gupta V, Brant J. Electronic Patient Symptom Management Program to Support Patients Receiving Cancer Treatment at Home During the COVID-19 Pandemic. Value in Health Elsevier; 2022 June 1;25(6):931–936. PMID:35339378

28. Lee M, Kang D, Kang E, Kim S, Kim Y, Ahn JS, Park S, Lee Y-Y, Oh D, Noh JM, Cho J. Efficacy of the PRO-CTCAE mobile application for improving patient participation in symptom management during cancer treatment: a randomized controlled trial. Support Care Cancer 2023 June;31(6):321. doi: 10.1007/s00520-023-07779-3

29. Girgis A, Bamgboje-Ayodele A, Rincones O, Vinod SK, Avery S, Descallar J, Smith A ‘Ben,’ Arnold B, Arnold A, Bray V, Durcinoska I, Rankin NM, the PROMPT-Care Implementation Authorship Group, Chang CF, Eifler B, Elliott S, Hardy C, Ivimey B, Jansens W, Kaadan N, Koh E-S, Livio N, Lozenkovski S, McErlean G, Nasser E, Ryan N, Smeal T, Thomas T, Tran T, Wiltshire J, Delaney GP. Stepping into the real world: a mixed-methods evaluation of the implementation of electronic patient reported outcomes in routine lung cancer care. J Patient Rep Outcomes 2022 Dec;6(1):70. doi: 10.1186/s41687-022-00475-6

30. Strasser F, Blum D, Von Moos R, Cathomas R, Ribi K, Aebi S, Betticher D, Hayoz S, Klingbiel D, Brauchli P, Haefner M, Mauri S, Kaasa S, Koeberle D. The effect of real-time electronic monitoring of patient-reported symptoms and clinical syndromes in outpatient workflow of medical oncologists: E-MO AIC, a multicenter cluster-randomized phase III study (SAKK 95/06). Annals of Oncology 2016 Feb;27(2):324–332. doi: 10.1093/annonc/mdv576

31. Maguire R, Ream E, Richardson A, Connaghan J, Johnston B, Kotronoulas G, Pedersen V, McPhelim J, Pattison N, Smith A, Webster L, Taylor A, Kearney N. Development of a Novel Remote Patient Monitoring System: The Advanced Symptom Management System for Radiotherapy to Improve the Symptom Experience of Patients With Lung Cancer Receiving Radiotherapy. Cancer Nursing 2015 Mar;38(2):E37–E47. doi: 10.1097/NCC.0000000000000150

32. Cox A, Illsley M, Knibb W, Lucas C, O’Driscoll M, Potter C, Flowerday A, Faithfull S. The acceptability of e-technology to monitor and assess patient symptoms following palliative radiotherapy for lung cancer. Palliat Med 2011 Oct;25(7):675–681. doi: 10.1177/0269216311399489

33. Gustafson DH, DuBenske LL, Atwood AK, Chih M-Y, Johnson RA, McTavish F, Quanbeck A, Brown RL, Cleary JF, Shah D. Reducing Symptom Distress in Patients With Advanced Cancer Using an e-Alert System for Caregivers: Pooled Analysis of Two Randomized Clinical Trials. J Med Internet Res 2017 Nov 14;19(11):e354. doi: 10.2196/jmir.7466

34. Zylla DM, Gilmore GE, Steele GL, Eklund JP, Wood CM, Stover AM, Shapiro AC. Collection of electronic patient-reported symptoms in patients with advanced cancer using Epic MyChart surveys. Support Care Cancer 2020 July 1;28(7):3153–3163. doi: 10.1007/s00520-019-05109-0

35. Iivanainen S, Alanko T, Vihinen P, Konkola T, Ekstrom J, Virtanen H, Koivunen J. Follow-Up of Cancer Patients Receiving Anti-PD-(L)1 Therapy Using an Electronic Patient-Reported Outcomes Tool (KISS): Prospective Feasibility Cohort Study. JMIR Form Res 2020 Oct 28;4(10):e17898. doi: 10.2196/17898

36. Basch E, Deal AM, Kris MG, Scher HI, Hudis CA, Sabbatini P, Rogak L, Bennett AV, Dueck AC, Atkinson TM, Chou JF, Dulko D, Sit L, Barz A, Novotny P, Fruscione M, Sloan JA, Schrag D. Symptom Monitoring With Patient-Reported Outcomes During Routine Cancer Treatment: A Randomized Controlled Trial. JCO 2016 Feb 20;34(6):557–565. doi: 10.1200/JCO.2015.63.0830

37. Yount SE, Rothrock N, Bass M, Beaumont JL, Pach D, Lad T, Patel J, Corona M, Weiland R, Del Ciello K, Cella D. A Randomized Trial of Weekly Symptom Telemonitoring in Advanced Lung Cancer. Journal of Pain and Symptom Management 2014 June;47(6):973–989. doi: 10.1016/j.jpainsymman.2013.07.013

38. Berry DL, Hong F, Halpenny B, Partridge A, Fox E, Fann JR, Wolpin S, Lober WB, Bush N, Parvathaneni U, Amtmann D, Ford R. The electronic self report assessment and intervention for cancer: promoting patient verbal reporting of symptom and quality of life issues in a randomized controlled trial. BMC Cancer 2014 July 12;14(1):513. doi: 10.1186/1471-2407-14-513
