# Supplementary figures and images for: The Current Landscape of Remote Digital Symptom Monitoring for Patients With Lung Cancer: Scoping Review
Source: J Med Internet Res. 2026 Mar 24;28:e83666. doi: 10.2196/83666 (PMC13012230; doi:10.2196/83666)

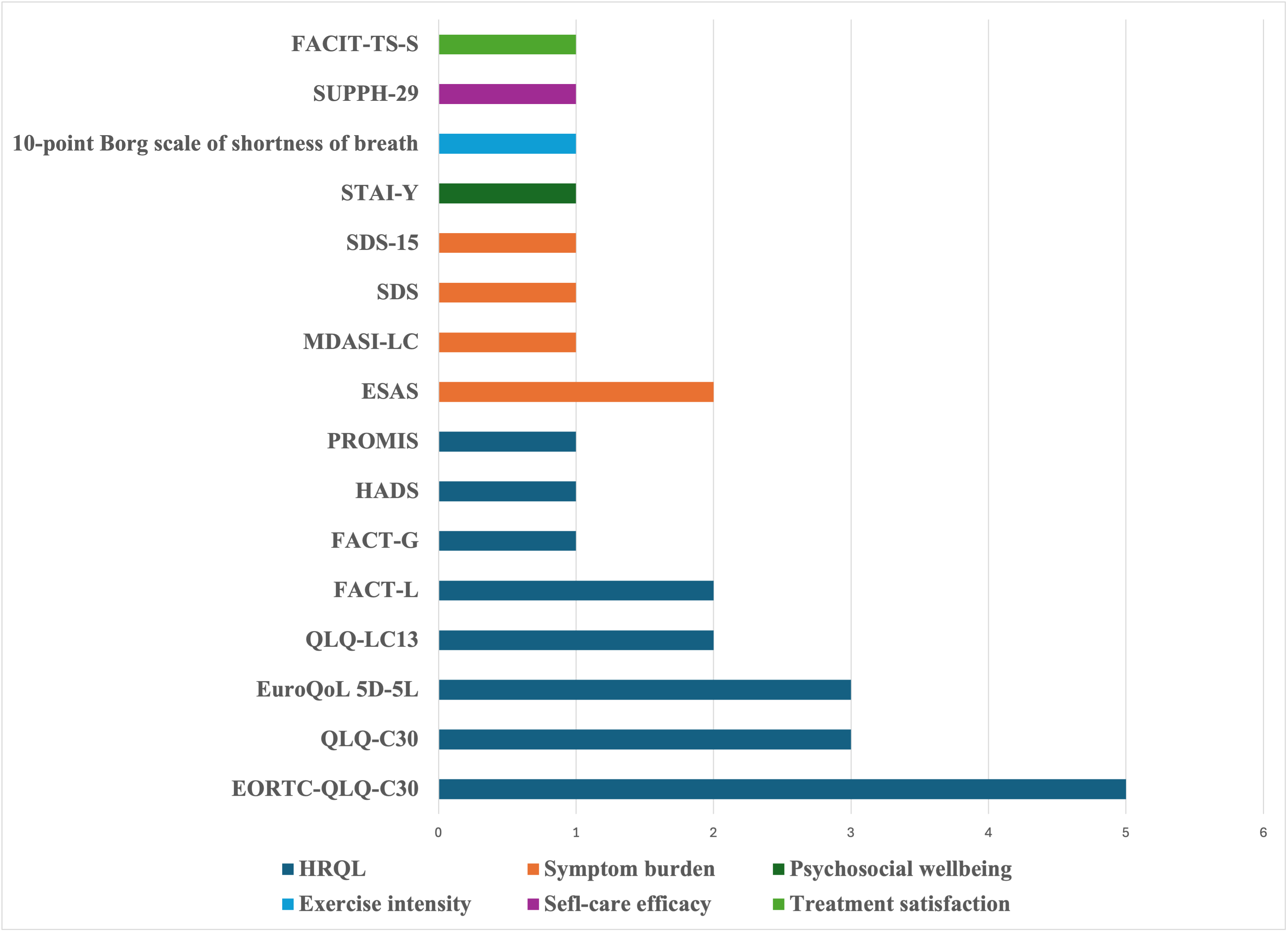

Supplement: Multimedia Appendix 4 [file jmir-v28-e83666-s004.png]
