# Supplementary material for: The Current Landscape of Remote Digital Symptom Monitoring for Patients With Lung Cancer: Scoping Review
Source: J Med Internet Res. 2026 Mar 24;28:e83666. doi: 10.2196/83666 (PMC13012230; doi:10.2196/83666)
Supplement: Multimedia Appendix 5 [file jmir-v28-e83666-s005.docx]

**Appendix 4 Content analysis of qualitative studies**

HCP’s perception of rSMS implementation

| Domain | Themes | Quotes |
| --- | --- | --- |
| Advantages | Empower the patient | I trust you [the patient], you tell me when you’ve got a problem, you tell me how you’re getting on, here’s this piece of technology to enable you to do that [1] |
|  |  | You could end up with a really good diary of how things have been over a week which you could then use to manage their pain more appropriately[1] |
|  |  | The best thing I think was the self-care advice because it's encouraging patients with their own health and to try things first cause that's what we would do for them anyway ... so I think it is putting the ball back in their court for their health em and you know trying that first and if they feel better after a self-help tip that they've done themselves, it gives them a bit of kind of encouragement to[2] |
|  | rSMS makes HCP better prepared | “... em, I think you, even in any situation so that you're, like a bit more prepared, em seeing what their, their issues are em, because they might have kind of forgotten what they put in to their questionnaire when you, when you phone them, em so it's just kind of saying you phoned and it makes them feel that you've actually ... it's very individual to them you've taken the time to look, you've taken the time to prepare before you phone so you've got the knowledge, and they don't need to go through everything again with you cause they might no [not] be feeling up to that”[2] |
|  | identify the relevant symptoms | Well, if they cough up blood, then it is absolutely certain, isn't it? If they have increasing shortness of breath, increasing fatigue, lack of appetite it looks like progression, right? Or if they have more than one symptom. So, in lung cancer patients, it's often the first thing we see.[3] |
|  |  | These are patients that we’re already getting fairly frequent monitoring...I do feel like it probably could be particularly helpful in some of our earlier stage patients that are not monitored except for every 3 or 6 months with scans. This may be helpful to kind of identify concerning symptoms and prompt and earlier evaluation.[4] |
|  | Identify the deterioration | Well, it could be both. We’ve had one (a patient) where there was a slow, creeping deterioration, and we think: “No, this can’t go on.“ Then it turned out that this patient had an appointment in the outpatient clinic, and there proved to be progression as well. But it was a creeping deterioration, actually. But it could be both, I think. It can easily go very quickly. [3] |
|  | Monitor condition changes | Well, there’s a kind of development here, isn't there? If the patient now has dyspnea and it's something the patient has experienced before, or is it something completely new, and if the patient has suddenly developed pain or has had pain at another time. It gives a better picture of an individual answers that can be compared with the others earlier answers. [3] |
|  | Assist clinical decision | And then you also have the security of knowing that there will be an answer again in a week. This means that you can continually evaluate. I then write to my colleagues in Cosmic (the electronic health record) if I'm not there the week after, “I have talked to the patient about this and that.“ I even had one (a patient) where I said you can just make a note in the comments at the next assessment regarding whether there’s been an effect. So, it can also be used as an evaluation tool. [3] |
|  |  | So, if we think that there could be progression, then we move the CT scan forward. We haven’t done this a lot because when we have suspected progression, a planned scan has usually been scheduled within a week's time, so we haven’t moved it forward, but if we suspect progression, we speed up the process in relation to when a scan should have taken place. [3] |
|  |  | Sometimes you help them get something more effective against nausea and pain; it can be advice and guidance about appetite and diet and what they should eat, and what they can do to be less tired. Some (patients) score high for pain, but where it turns out that they are massively constipated, so it can be advice about constipation and things like that. [3] |
|  |  | I am happy to have been involved. I think that it has a time and a place for the portal. I think it’s just kind of like finding the right population here to use it in. I definitely think that patient-reported outcomes are extremely important and useful, you know, to kind of guide our practice. If this was implemented in the appropriate patient population, it would be really helpful.[4] |
|  | Relax patients | I don't feel that distance is a problem; on the contrary, I think that the fact that they are at home in their own familiar surroundings and nobody else is listening makes them speak a little more freely than they would if they were sitting in a treatment room. So, I also have conversations with them about existential issues, which I probably wouldn't be able to have if they were here in the clinic because there are so many people sitting around. [3] |
| Disadvantages | rSMS should be complement rather than replace clinical judgement | I think they can be useful for a baseline but I think what they shouldn’t do is replace the experience of the person making the assessment. [1] |
|  |  | I think there has always got to be scope for looking at that particular patient and looking at their own specific needs in maybe a slightly different. . .much more holistic way than that tool allows[1] |
|  |  | When you cannot see the patient in person, you at least need to have some experience in asking about problems and symptoms. It is important that you have knowledge of the specialty and have the possibility to know what to ask to get the right information out of the patient.[3] |
|  |  | They (doctors) don't see anything unless I show it to them, and it's always a balancing act between not wanting to bother the doctors unnecessarily with something that’s not important, but then again, you don’t want to overlook anything. [3] |
|  | Tech limited to older population | Age is a factor actually, a strong factor in terms of kind of getting our heads around something like this, something that the very elderly, and I am generalising now but something the very elderly just didn’t want anything to do with[1] |
|  |  | I think the hub would probably be much more appropriate for say more. . .perhaps your younger cohort of patients[1] |
|  |  | I think the majority of the patients that we look after are still in a group age wise that isn’t that familiar with e-technology so that’s a potential problem[1] |
|  | Careful when implementing the rSMS in specific care population | Because of the nature of palliative care patients things move quite quickly. . ..and they can become unwell quite rapidly. . .and I think trying to get to grips with using a new piece of technology if somebody was very unwell or symptomatic might be tricky[1] |
|  |  | it is probably beneficial for some patients but I think if you’ve got patients who are highly anxious you could be getting alerted a lot of the time for lots of reasons that really aren’t necessary[1] |
|  |  | Well it’s just an emotional, ethical. . .you know they’ve already got such huge burden, why burden, how do we dare burden them with anything else[2] |
|  |  | I think we always feel protective of people if they are really very, very unwell. . .you think about research generally and whether it’s an added burden for them rather than a benefit to them[2] |
|  |  | I think really kind of thinking about the questions that patients are [being] asking and then tailoring them a little bit to the populations would be 1 of my major points.[4] |
|  | Lack of face to face contact | I don’t think you could beat the sort of personal face to face or telephone contact[1] |
|  |  | Some people might feel quite anxious about it or feel that it’s a bit depersonalising[1] |
|  |  | And that is one of the things that worries me about all this technol. . ..All technology not just this is it gets you further and further away from clinically looking at the patient and thinking you actually don’t look well[1] |
|  |  | Well, it’s very difficult to assess a patient without using your clinical eyes. You have to ask some challenging questions to understand what’s going on. But in any case, it’s the clinical gaze that’s best if it is possible to use it. [3] |
|  | Increase workload | It takes the patient to key-in something and then for a nurse to respond to an additional piece of equipment that they have to carry apart from the equipment they already carry. It then depends on them picking that that alert up and then physically logging into another system in addition to the system they will always be logging into and then phoning the patient. So compared to the system that I'm used to, it seems cumbersome, it adds in too many other things to do to actually get to the patient. [2] |
|  |  | Sometimes, someone else in the group has a little extra time and comes in and says, “Well, I'll take the ProWide responses,” and then it's okay, but the responsibility for whether it is done lies with the coordinator. And if we've all been very busy, then I'll just go in and look through how many there are; well, you know, then I just open them up and see if there's anything that simply needs to be taken care of; otherwise they wait until the next day. [3] |
|  | Burden of alerts (Time/frequency) | ...like if we were trying to track how patients receiving active treatment for lung cancer symptoms change over time then the ... portal would have been very relevant...[4] |
|  | Alert utility | ...it would be great if all alerts were clinically relevant. If there was a way to ensure that whatever alert came from the patient was because the provider really needed to do something...[4] |
|  | Insufficient information | Yes, that's the disadvantage of PRO. You can use a questionnaire with answers, but that doesn't say anything about how the patient is actually doing. So it can be difficult to conclude from an answer how the patient is feeling. You have to have a few more words from the patient, for example, when did you start coughing and what kind of cough is it, how does the cough affect you? These are questions that you need to get answered in connection with the questionnaire itself.[3] |
|  |  | I think talking to the patient is crucial; you have to elaborate on something there, right? For example, if there is blood and how much blood there is, and how often is there blood. The same with increased difficulty of breathing; when did it happen? Do you cough? Do you have stridor? So, some things have to be done more in-depth than can be done in a questionnaire when considering disease progression. There is a need for an in-depth conversation. Yes, there is[3] |
|  | Accuracy in question | I think the most difficult is when the person scores 'red’ for the third week in a row and just stays the same and doesn't budge, but that’s the way it is. And there’s the purely psychological aspect of it, the thing about knowing that the usual state of a person in question is to be dyspneic or have a cough or something else, that it's just the usual thing, and I can't do anything about it. And that’s the “helper gene“ again; you want to solve all their problems, or at least those problems that you think you should be able to solve[3] |
|  |  | And there are also a lot of people out here who underreport; I think just a little, at least, right? So sometimes it can also be interesting to get a hold of them and hear what something is really all about, right? And how bad is it? ... Can you do what you usually do? Things like that. And there are also some of them who just say, well, I feel bad, without actually being able to put it into words. Some men don’t have a lot of words. And I think you should be worried about them. [3] |
|  | Provider training is useful | Well, I guess [the training] just needs to be more in-depth. ....it just maybe needs to be more specific, I guess, and less broad.[4] |
|  | Should be integrated in EHR | I think in theory it could [have been useful]. It sounds like it could be useful, however...the communication is more clunky than it would be if the patient just reached out via the medical record.[4] |

Patients’ perception of rSMS implementation

| Domain | Themes | Quotes |
| --- | --- | --- |
| Advantages | Easy to use | I had no hassle with it at all, and as I say having, you know I'm no(t) great on computers and things but I have some knowledge, so I was able to do it[2] |
|  |  | [The surveys] weren’t that long, so they weren’t time consuming at all.[5] |
|  |  | The questions and the answers were self-explanatory. It was pretty simple, pretty easy to answer the questions. [5] |
|  |  | They don’t take that long to fill out. I didn’t have any issues. It was just easy. I Would breeze through it. [5] |
|  | Helpful and instant | Well as far as I am concerned yes, because it was very helpful because I had this bad cough and one or two alerts came up and the nursing staff at the other end were immediately onto it... the fact that we were in contact with the hospital very much quicker than we would be if we’d waited and maybe even phoned[2] |
|  |  | Oh it was helpful, yes ... yes, that was helpful. Aye well the, the coughing and just to, reminding you to lie upright ... upright, that was the thing you’re inclined to forget ..... you know to sort of eh, even sit up, up in bed ... pillows up and eh coughing, and what was the other thing ... ? breathless, breathless, instead of panicking, sometimes you could be inclined to, if you just let it get on top of you, and being reassured there that just, to do your breathing exercises which I had got in the hospital ... and just relax, so that, that, that was good[2] |
|  |  | That would have been helpfuldyes, that the doctor, the nurse practitioner, all the different people that you’ve met along this journey [introduce the study]. [5] |
|  | Perceived Clinical Utility and Connectedness | “When I arrived here, I felt more prepared and calm. It seemed that between visits, there was no interruption. I felt followed even at home.”(P5))  “I had the feeling of arriving more prepared for meetings with my doctors. I saw it as a liaison rather than a tool in itself. I felt involved in my illness; there were targeted questions.(P5) [6] |
|  | Emotional Anchoring and Psychological Reassurance | I think it’s a necessity almost, it just keeps your morale up and I think it’s a great little gadget. I’m glad, let me say I’m glad I had it and I’m glad I used it[2] |
|  |  | I was very pleased because once you’re away from the hospital and you needed contact with them you’re out on a limb sort of thing.....and you do tend to think “oh well perhaps this isn’t anything” and at least when you’re in contact with them (via ASyMS) they can, they know whether it is anything that’s necessary or not. So yes I quite agree with it, I’m glad that they were (there) [2] |
|  |  | I think it’s beneficial because it makes you think about how you’re feelin’. Again, I was goin’ through so much that I think it was crucial, really, to make sure there wasn’t major problems with me. [5] |
|  |  | [The surveys help] you keep up with yourself, plus you feel like you have support from another . source[5] |
|  |  | “Having an app that is managed and supervised by your doctors makes you feel secure because you know there is a connection. You know they check and review the answers; if something is wrong, they call you. This makes you feel constantly safe, anchored to the place where you are treated, where you feel protected.”(P2)  “the app was almost like a companion for me, if I may say so. I found the questions dynamic, I liked how they were formulated. The need to answer these questions was almost a stimulus. I did it in the morning during breakfast; it became a routine for me to start the day. These questions stimulated me(P3)[6] |
|  | Cognitive Engagement and Self-Reflection | “Well, reflecting on all these aspects every day, in my opinion, helps to downplay things a bit. Of course, one day might be more negative, another more positive, but reflecting prevents marking the worst level because you reason better, helping to be more tranquil” (P6)  I used to have the attitude of 'I don't think about it, and it will pass.' But having to answer the app’s questions every day gave me a different kind of awareness—it helped me recognize and manage my symptoms better, day by day. It became a crucial tool for connecting with my body in a way I hadn’t before." .(P4))[6] |
|  | Integration into Daily Life and Routines | What I can say is that having the app gave me a sense of protection. In the morning, encountering the app lightened my day, even in the heaviest periods. It opened a more favorable door to the day for me. It's a very valid tool for cancer patients because there are various psychological aspects it can help with.[6] |
|  | Altruistic Motivation and Contribution to Others | “It felt good to know that by using the app, I wasn’t just helping myself—I was also helping others who might go through the same thing."(P1)[6] |
| Disadvantages | Limited by health condition | There was days where I was feeling awful and I didn’t complete the survey, but it was because of how I was physically feeling, not because of I didn’t wanna do the survey, if that makes sense.[5] |
|  |  | I think I was just mainly exhausted, and it was that [the surveys] were helpful and everything, but ... [5] |
|  |  | ... a couple of weeks that I didn’t complete [the surveys] . was at the beginning because I was still going through a little bit of, I guess, side effects from the surgery, so I really wasn’t doing a lot of things on the computer or on the internet at that time. That was the only time[5] |
|  | Technology issue | Well, my daughter, I was usin’ her email, and it wouldn’t let her login. see, my daughter do that, and she live [elsewhere], so I’d rather be called on the phone to answer any survey[5] |
|  |  | .I live in the country. If anything, the service is a little slow, but as far as loggin’ in and answerin’ the questions and stuff it was no problem. [5] |
|  |  | It’s just I tried to log in on my phone, and it wouldn’tdyou know, it just wouldn’t go to that website. [5] |
|  | Irrelevant or repeated ePRO monitoring questions | Aye, I'll be honest about that, I scanned over it [self-care] because I was getting, I, I was getting the treatment for it, do you know I was doing, what I should have been doing kind of thing it wis'nae [was not] anything that was new to me[2] |
|  |  | I think also because the surgery didn’t really turn out the way that it was expected, that I think a lot of the questions didn’t really apply to me because, like I said, [surgery] didn’t really do what it was supposed to do[5] |
|  |  | I think it was aimed [at someone with more extreme symptoms]dI came back negative for cancer. and I’m in overall good health. I don’t want to say [the surveys were] monotonous because youdI can see where the questions need to be asked and in the time frame that they’re asked. It was just for me, it was just asking a lot of questions that I wasn’t running into. [5] |

References

1. Cox A, Illsley M, Knibb W, Lucas C, O’Driscoll M, Potter C, Flowerday A, Faithfull S. The acceptability of e-technology to monitor and assess patient symptoms following palliative radiotherapy for lung cancer. Palliat Med 2011 Oct;25(7):675–681. doi: 10.1177/0269216311399489

2. Maguire R, Ream E, Richardson A, Connaghan J, Johnston B, Kotronoulas G, Pedersen V, McPhelim J, Pattison N, Smith A, Webster L, Taylor A, Kearney N. Development of a Novel Remote Patient Monitoring System: The Advanced Symptom Management System for Radiotherapy to Improve the Symptom Experience of Patients With Lung Cancer Receiving Radiotherapy. Cancer Nursing 2015 Mar;38(2):E37–E47. doi: 10.1097/NCC.0000000000000150

3. Schougaard LMV, Friis RB, Grytnes R, Grove BE, Hjollund NH, Pappot H, Skuladottir H, Mejdahl CT. Exploring the Nurses’ Perspective on Using Remote Electronic Symptom Monitoring in Clinical Decision-Making Among Patients With Metastatic Lung Cancer. Seminars in Oncology Nursing 2023 Dec;39(6):151517. doi: 10.1016/j.soncn.2023.151517

4. Stover AM, Deal AM, Medley CJ, Weiner AA, Novak L, Gentry AL, Hoch C, Weiss J, Pecot CV, Lee CB, O’Leary MC, Shrestha S, Chen H, Patel SA, Mody GN. Feasibility, Acceptability, and Utility of Remote Patient-Reported Outcomes Monitoring in Patients With Lung Cancer: A Moovcare© Study. Clinical Lung Cancer 2025 Aug;S1525730425001603. doi: 10.1016/j.cllc.2025.07.016

5. Boisson-Walsh A, Cox C, O’Leary M, Shrestha S, Carr P, Gentry AL, Hill L, Newsome B, Long J, Haithcock B, Stover AM, Basch E, Leeman J, Mody GN. A Qualitative Study of Electronic Patient-Reported Outcome Symptom Monitoring After Thoracic Surgery. Journal of Surgical Research 2024 Nov;303:744–755. doi: 10.1016/j.jss.2024.09.051

6. Pongiglione B, Cucciniello M, Petracca F, Ciani O, Novello S, Migliorino M, Pedrazzoli P, Agustoni F, Lo Russo G, Tarricone R, Capelletto E. A mobile supportive care app for patients with metastatic lung cancer: the Lung Cancer App (LuCApp) randomized controlled trial. Support Care Cancer 2025 July;33(7):641. doi: 10.1007/s00520-025-09682-5
