# Supplementary material for: The Current Landscape of Remote Digital Symptom Monitoring for Patients With Lung Cancer: Scoping Review
Source: J Med Internet Res. 2026 Mar 24;28:e83666. doi: 10.2196/83666 (PMC13012230; doi:10.2196/83666)
Supplement: Checklist 2 [file jmir-v28-e83666-s007.pdf]

PRISMA-S Checklist

Section/topic	#	Checklist item	Location(s) Reported
**INFORMATION SOURCES AND METHODS**			
Database name	1	Name each individual database searched, stating the platform for each.	Page 6 Line 65-69
Multi-database searching	2	If databases were searched simultaneously on a single platform, state the name of the platform, listing all of the databases searched.	NA
Study registries	3	List any study registries searched.	NA
Online resources and browsing	4	Describe any online or print source purposefully searched or browsed (e.g., tables of contents, print conference proceedings, web sites), and how this was done.	NA
Citation searching	5	Indicate whether cited references or citing references were examined, and describe any methods used for locating cited/citing references (e.g., browsing reference lists, using a citation index, setting up email alerts for references citing included studies).	Page 7 Line 75-76
Contacts	6	Indicate whether additional studies or data were sought by contacting authors, experts, manufacturers, or others.	Page 7 Line 76-79
Other methods	7	Describe any additional information sources or search methods used.	NA
**SEARCH STRATEGIES**			
Full search strategies	8	Include the search strategies for each database and information source, copied and pasted exactly as run.	Appendix 1
Limits and restrictions	9	Specify that no limits were used, or describe any limits or restrictions applied to a search (e.g., date or time period, language, study design) and provide justification for their use.	Page 7 Line 73-74
Search filters	10	Indicate whether published search filters were used (as originally designed or modified), and if so, cite the filter(s) used.	Page 7 Line 73-74
Prior work	11	Indicate when search strategies from other literature reviews were adapted or reused for a substantive part or all of the search, citing the previous review(s).	NA
Updates	12	Report the methods used to update the search(es) (e.g., rerunning searches, email alerts).	Page 7

			Line 80
Dates of searches	13	For each search strategy, provide the date when the last search occurred.	Page 7<br>Line 80
**PEER REVIEW**			
Peer review	14	Describe any search peer review process.	Page 6<br>Line 69-71
**MANAGING RECORDS**			
Total Records	15	Document the total number of records identified from each database and other information sources.	Figure 1
Deduplication	16	Describe the processes and any software used to deduplicate records from multiple database searches and other information sources.	Page 8<br>Line 100-101

PRISMA-S: An Extension to the PRISMA Statement for Reporting Literature Searches in Systematic Reviews
Rethlefsen ML, Kirtley S, Waffenschmidt S, Ayala AP, Moher D, Page MJ, Koffel JB, PRISMA-S Group.
Last updated February 27, 2020.
